# tRNA-derived fragments in T lymphocyte–beta cell crosstalk and in type 1 diabetes pathogenesis in NOD mice

**DOI:** 10.1007/s00125-024-06207-3

**Published:** 2024-07-05

**Authors:** Flora Brozzi, Cécile Jacovetti, Cristina Cosentino, Véronique Menoud, Kejing Wu, Mustafa Bilal Bayazit, Baroj Abdulkarim, Christian Iseli, Nicolas Guex, Claudiane Guay, Romano Regazzi

**Affiliations:** 1 Department of Fundamental Neurosciences, University of Lausanne, Lausanne, Switzerland; 2 Haya Therapeutics SA, Épalinges, Switzerland; 3 Bioinformatics Competence Centre, University of Lausanne, Lausanne, Switzerland; 4 Bioinformatics Competence Centre, EPFL, Lausanne, Switzerland; 5 Department of Biomedical Sciences, University of Lausanne, Lausanne, Switzerland

**Keywords:** Apoptosis, Autoimmunity, Extracellular vesicles, Insulin, Pancreatic islet

## Abstract

**Aims/hypothesis:**

tRNAs play a central role in protein synthesis. Besides this canonical function, they were recently found to generate non-coding RNA fragments (tRFs) regulating different cellular activities. The aim of this study was to assess the involvement of tRFs in the crosstalk between immune cells and beta cells and to investigate their contribution to the development of type 1 diabetes.

**Methods:**

Global profiling of the tRFs present in pancreatic islets of 4- and 8-week-old NOD mice and in extracellular vesicles released by activated CD4^+^ T lymphocytes was performed by small RNA-seq. Changes in the level of specific fragments were confirmed by quantitative PCR. The transfer of tRFs from immune cells to beta cells occurring during insulitis was assessed using an RNA-tagging approach. The functional role of tRFs increasing in beta cells during the initial phases of type 1 diabetes was determined by overexpressing them in dissociated islet cells and by determining the impact on gene expression and beta cell apoptosis.

**Results:**

We found that the tRF pool was altered in the islets of NOD mice during the initial phases of type 1 diabetes. Part of these changes were triggered by prolonged exposure of beta cells to proinflammatory cytokines (IL-1β, TNF-α and IFN-γ) while others resulted from the delivery of tRFs produced by CD4^+^ T lymphocytes infiltrating the islets. Indeed, we identified several tRFs that were enriched in extracellular vesicles from CD4^+^/CD25^−^ T cells and were transferred to beta cells upon adoptive transfer of these immune cells in NOD.SCID mice. The tRFs delivered to beta cells during the autoimmune reaction triggered gene expression changes that affected the immune regulatory capacity of insulin-secreting cells and rendered the cells more prone to apoptosis.

**Conclusions/interpretation:**

Our data point to tRFs as novel players in the crosstalk between the immune system and insulin-secreting cells and suggest a potential involvement of this novel class of non-coding RNAs in type 1 diabetes pathogenesis.

**Data availability:**

Sequences are available from the Gene Expression Omnibus (GEO) with accession numbers GSE242568 and GSE256343.

**Graphical Abstract:**

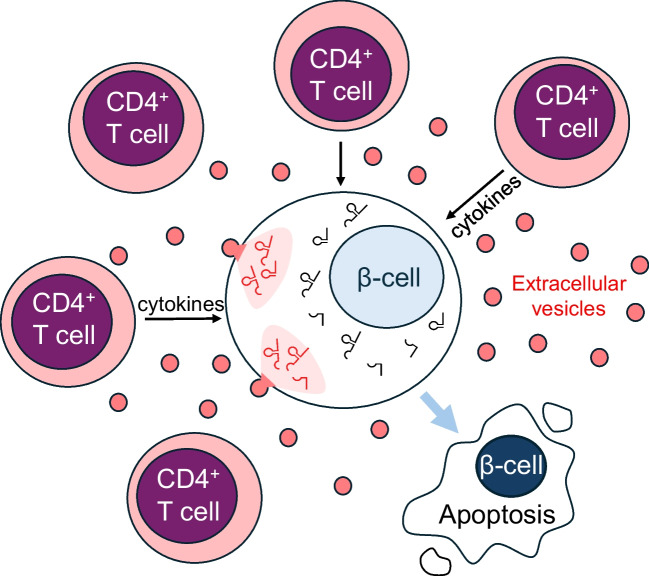

**Supplementary Information:**

The online version of this article (10.1007/s00125-024-06207-3) contains peer-reviewed but unedited supplementary material.


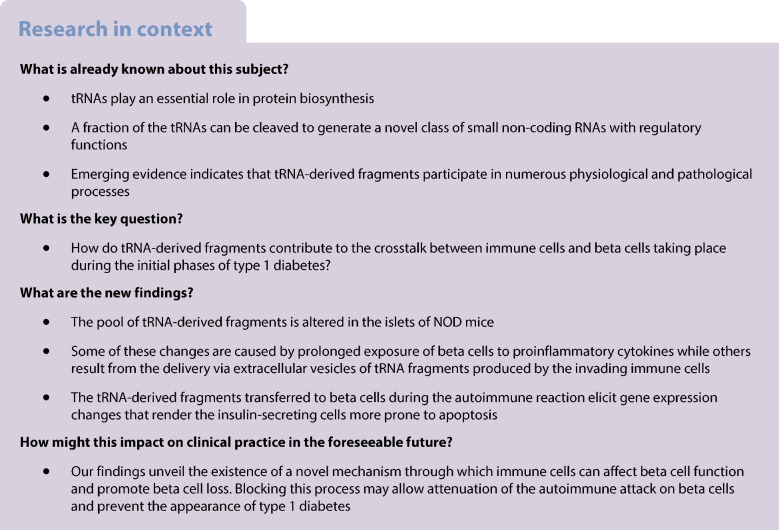


## Introduction

Type 1 diabetes is characterised by the invasion of the islets of Langerhans by immune cells and by an autoimmune attack against insulin-secreting beta cells. The autoimmune reaction culminates in a near-complete elimination of beta cells, resulting in chronic hyperglycaemia and in the development of diabetes mellitus [1, 2]. During islet invasion, the infiltrating immune cells release proinflammatory cytokines that cause beta cell dysfunction and promote beta cell apoptosis [3]. Beside these inflammatory mediators, the invading immune cells release extracellular vesicles (EVs) that contribute to beta cell dysfunction and diabetes development [4].

Non-coding RNAs are regulators of cellular activities and are involved in many human diseases, including diabetes [5–8]. The expression of different classes of non-coding RNAs present in insulin-secreting cells is altered during the initial phases of type 1 diabetes and participates in beta cell failure. These changes are caused in part by exposure of insulin-secreting cells to proinflammatory cytokines, while others may result from the delivery of RNAs carried by EVs released by the invading immune cells.

tRNA-derived fragments (tRFs) constitute a recently described group of non-coding RNAs regulating numerous cellular functions [9, 10] and originate from the cleavage of precursors or mature tRNAs [9, 11]. tRNA cleavage may be elicited in response to external stimuli [12, 13] and depends on the accessibility of the cleavage site, which is modulated by the presence of nucleotide modifications [14] and/or the association of RNA-binding proteins [15, 16]. Depending on the cleavage site, this process can yield different types of fragments that are classified according to their length and the region of the tRNA to which they map [17].

We recently demonstrated that beta cells contain a specific pool of tRFs and that some of them play an important role in neonatal beta cell mass expansion and the acquisition of a fully functional secretory phenotype [18]. In this study, we investigated whether changes in tRF level contribute to beta cell failure during the initial phases of type 1 diabetes, favouring the appearance of the disease.

## Methods

### Reagents

The sources of the reagents and the animals used in this study can be found in electronic supplementary material (ESM) Table 1.

### Cell lines

Cell lines were regularly tested for mycoplasma contamination. Jurkat T cells [19] were cultured in RPMI 1640 medium supplemented with 10% (vol./vol.) EV-depleted FBS, 50 μg/ml streptomycin and 50 IU/ml penicillin. MIN6B1 cells [20], were cultured in DMEM-GlutaMAX high-glucose medium supplemented with 15% (vol./vol.) FBS (depleted of EVs for the experiments assessing the transfer of tRF), 70 μmol/l β-mercaptoethanol, 50 μg/ml streptomycin and 50 IU/ml penicillin.

### Animals

Male C57BL/6NRj mice (aged 12 weeks), female NOD.CB-17-Prkdc scid/Rj mice (aged 11–13 weeks), female NOD/ShiLtJ (aged 4 weeks and 8 weeks) and female NOD.Cg-Tg (TcraBDC2.5, TcrbBDC2.5)1Doi/DoiJ, HEM mice (aged 8 weeks) were housed under a 12 h light–dark cycle.

### Islet isolation, cell dissociation and beta cell sorting

Mouse pancreases were digested with collagenase and islets were isolated by Histopaque density gradient [21]. Islet cells were cultured in RPMI 1640 GlutaMAX medium supplemented with 10% (vol./vol.) FBS, 100 U/ml penicillin, 100 µg/ml streptomycin, 1 mmol/l sodium pyruvate and 10 mmol/l HEPES. When needed, rodent islets were dissociated by incubation in Ca^2+^/Mg^2+^-free PBS, 3 mmol/l EGTA and 0.002% (vol./vol.) trypsin for 2–3 min at 37°C.

Fractions enriched of beta cells were obtained by FACS of dissociated islet cells based on autofluorescence [22]. Dissociated islet cells were washed once with FACS buffer (0.1% (wt/vol.) BSA, 2 mmol/l EDTA, 11 mmol/l glucose in PBS) and exposed for 5 min to TruStain FcX (anti-mouse CD16/32) Antibody at 4°C. Then cells were incubated for 30 min in the dark at 4°C with the following antibodies: 1:200 of FITC anti-CD45, 1:40 of allophycocyanin anti-CD3, 1:20 of Brilliant Violet 421 anti-CD4 and 1:20 of phycoerythrin anti-CD25. Cells were washed twice with FACS buffer and analysed by FCF-Aria-II SORP. Beta cell purity was assessed as previously described [4]. Beta cell fractions contained 99.1 ± 0.9% insulin-positive cells and 0.6 ± 0.6% glucagon-positive cells.

### Isolation, culture and EU-labelling of T lymphocytes

Mouse CD4^+^/CD25^−^ T cells were purified from lymphoid tissues of NOD/ShiLtJ and NOD.Cg-Tg (TcraBDC2.5, TcrbBDC2.5) mice, using CD4^+^CD25^+^ Regulatory T Cell Isolation Kit. CD4^+^/CD25^–^ T cells were incubated in RPMI 1640 medium supplemented with 10% EV-depleted FBS, 100 μg/ml streptomycin and 100 IU/ml penicillin and were stimulated for 72 h with 20 ng/ml IL-12, 200 U/ml IL-2 and Dynabeads Mouse T-Activator CD3/CD28 beads, or 2 µg/ml of anti-CD28 and 5 µg/ml of anti-CD3 for NOD.BDC2.5 T cells. When needed, T cells were treated with 200 µmol/l 5-ethynyl uridine (EU) during the last 48 h, before EV and RNA collection.

### RNA extraction and quantitative PCR

Total RNA was extracted with the miRNeasy kit. Real-time PCR quantification of tRFs and miRNAs was performed using the miRCURY LNA Universal RT microRNA PCR system. The sequences used for custom primer design are indicated in ESM Table 2. For gene expression quantification, RNA was treated with DNase and reverse transcribed with a Moloney Murine Leukemia Virus reverse transcriptase and random primers. Quantitative PCR (qPCR) was performed using SsoAdvanced Universal SYBR Green Supermix. Primer sequences are listed in ESM Table 1. Expression of miRNAs, tRFs and protein-coding genes was corrected for the level of miRNAs and housekeeping genes that are unaffected by the experimental condition under study.

### Small RNA-seq

RNA was extracted with miRNeasy kit and pretreated with rtStar tRF&tiRNA Pretreatment Kit to remove tRNA modifications. Libraries were prepared with the QIAseq miRNA Library Kit and analysed using the Agilent BioAnalyzer 2100. The samples were then sequenced with an Illumina NextSeq 500 instrument.

### tRF annotation and analysis

We used fastx toolkit 0.0.13 (http://hannonlab.cshl.edu/fastx_toolkit/) to clip the adapter sequences. Identical reads longer than 16 nts were collapsed based on Unique Molecular Identifiers and aligned to the mouse genome (GRCm38.p6). Aligned reads were mapped to the mature tRNA sequences using bowtie (version 1; http://bowtie.cbcb.umd.edu). Bowtie parameters were set to output only perfect matches to tRNA sequences. The tRNA sequences were downloaded from the GtRNAdb database (Release 1.0; http://gtrnadb.ucsc.edu/, accessed 15 October 2020) and the 22 mouse mitochondrial tRNA sequences were retrieved from https://www.ncbi.nlm.nih.gov/nuccore/J01420.1 (accessed 15 October 2020).

tRFs displaying differential levels between the groups were identified using edgeR (version 4.2.2) in R packages (http://bioconductor.org). For each tRF, *p* values and false discovery rates (FDRs) were obtained based on the model of negative binomial distribution. Fold changes of gene expression were also estimated within the edgeR statistical package. The criterion for differential expression of tRFs has been set as fold change >2 and *p* value <0.05.

### EV isolation and quantification

EVs were isolated as previously described [4]. Briefly, the culture media of Jurkat cells or primary human or mice T cells were centrifuged at 300 *g* for 6 min and then at 2000 *g* for 10 min. Supernatant fractions were centrifuged at 10,000 *g* for 30 min to remove cell debris and then at 100,000 *g* for 2 h. The pellet containing the EVs was washed twice with PBS and re-centrifuged at 100,000 *g* for 2 h.

MIN6B1 or dispersed mouse islet cells were incubated in FBS-EV-depleted medium. The number of vesicles added to the cells was estimated using Zetasizer Ultra. The recipient cells were exposed to EV concentrations of about 1×10^11^/ml.

### T cell adoptive transfer, beta cell isolation and identification of EU-tagged RNA

CD4^+^/CD25^−^ T cells from NOD.Cg-Tg (TcraBDC2.5, TcrbBDC2.5) mice were isolated and activated as described above and treated for 48 h with 200 μmol/l of EU. The cells were washed with PBS and resuspended in sterile saline solution (154 mmol/l NaCl). Approximately 3–4×10^6^ T cells were injected in the tail vein of each NOD.CB-17-Prkdc scid/Rj mouse. Control mice were injected i.v. with the same number of T cells, not treated with EU (CTRL-2), or with the same volume of saline solution, without T cells (CTRL or ‘sham’). At 48 h after injection, mice were killed and islet cells isolated. The islets of three mice per condition were combined, cultured for 1 h and then dispersed and labelled with antibodies prior to FACS analysis. FAC-sorted beta cells were purified from the CD45-negative fraction based on beta cell autofluorescence. RNA was extracted with the miRNeasy kit, and the same amount of EU-tagged *Caenorhabditis elegans* miR-238 mimic was added to all the samples. EU-tagged RNA was biotinylated using the Click-iT Nascent RNA Capture kit and incubated with streptavidin-coated beads. The biotinylated RNA was eluted in a solution containing 95% formamide and 10 mmol/l EDTA. Eluted RNA was purified again with the miRNeasy kit and used for library preparation.

### RNA-seq analysis of pulled-down EU-tagged RNA

We wrote a Perl script to analyse the reads and extract the sequence of the captured RNAs (for details, see ESM Methods). Using this approach, we obtained 13,323,134 sequences, which were filtered to select only sequences of 16–55 nucleotides. The sequences with at least five counts in at least four samples and that originated from fragments of the tRNAs retrieved from http://gtrnadb.ucsc.edu/genomes/eukaryota/Mmusc39/ or fragments of the control miR-238 *C. elegans* sequence were retained and analysed with R (version 4.2.2) and DEseq2 (version 1.38.3 [23]) with default parameters.

### Cell transfection and treatment

For tRF overexpression, dissociated mouse islet cells were transfected using Lipofectamine 2000 with control oligonucleotides or tRF mimics designed from the sequences in ESM Table 2. For tRF inhibition, cells were transfected with a control or custom-designed tRF inhibitors against the sequences in ESM Table 2. Cells were cultured for 48 h before RNA extraction or functional assays. To assess the impact of proinflammatory cytokines, islet cells were incubated for 24 h, with IL-1β (0.1 ng/ml), TNF-α (10 ng/ml) and IFN-γ (30 ng/ml), or with IFN-α (1000 U/ml).

### Beta cell apoptosis

The percentage of dying cells was determined after staining with propidium iodide (5 μg/ml; Sigma) and Hoechst 33342 (5 μg/ml). A minimum of 1000 cells was blindly counted for each experimental condition. The percentage of beta cells undergoing apoptosis was assessed after fixation with methanol, permeabilisation with saponin and double-staining with guinea pig anti-insulin and rabbit anti-cleaved caspase-3 antibodies. The coverslips were then incubated with Alexa 488 anti-guinea pig and Alexa 555 anti-rabbit antibodies for 1 h at room temperature. The coverslips were incubated with Hoechst dye 33342 and mounted with Fluor-Save mounting medium. Pictures were taken using a Zeiss Axiovision fluorescence microscope. A minimum of 600 cells was counted blindly for each experimental condition.

### mRNA sequencing

Libraries were prepared with the TruSeq Stranded mRNA Library Prep kit and sequenced with a HiSeq 2500 instrument using the SBS chemistry v4 (Illumina). Differentially expressed genes were identified using the Qiagen RNAseq analysis platform: QIAGEN RNA-seq Analysis Portal 4.1. Normalisation and differential gene expression (FDR<0.05) were performed using DESeq2 (v.1.12.4).

### Statistical analysis

No data were excluded from the study and no randomisation was carried out. Data are presented as the mean ± SD. A two-tailed one-sample *t* test was used to compare datasets to a control value settled to 1, and two-tailed paired or unpaired *t* tests were used for comparing two datasets. One- or two-way ANOVA followed by Dunnett or Sidàk post hoc tests were used to compare multiple datasets. Correlation of datasets was assessed by a two-tailed Pearson test. Differences between datasets were considered significant when the *p* value was <0.05.

### Gene Expression Omnibus submissions

Sequences are available from the Gene Expression Omnibus (GEO) (https://www.ncbi.nlm.nih.gov/geo/) with accession numbers GSE242568 and GSE256343.

## Results

### The pool of tRNA-derived fragments is altered in NOD mouse islet cells during insulitis

**Fig. 1:**
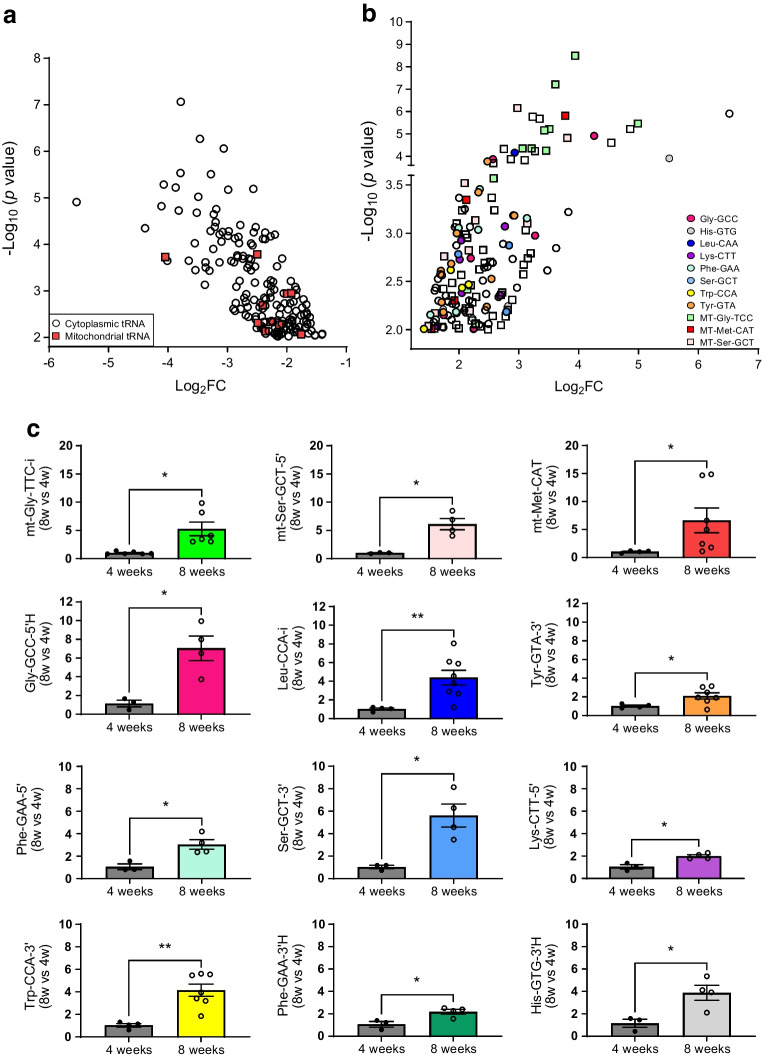
Identification of tRFs displaying changes in their level in the islets of prediabetic NOD mice. (**a**) tRFs significantly downregulated in the islets of 8-week-old mice compared with the islets of 4-week-old mice. (**b**) tRFs significantly upregulated in islets of 8-week-old mice compared with the islets of 4-week-old mice. Squares, tRFs derived from mitochondrially encoded tRNAs; circles, cytoplasmic tRFs. Colour codes indicate fragments originating from the same isoacceptor tRNA. (**c**) Real-time PCR confirmation of the upregulation of selected tRFs in islets of 8-week-old mice. The data were normalised to the level of the small ncRNA miR-184–5p, which displays no changes under prediabetic conditions. The level of the fragments in 4-week-old mice has been set to 1 and the data shown are the means ± SD. *n*=3–7. *p<0.05, **p<0.01 (unpaired *t* test). FC, fold change

NOD mice constitute the best characterised animal model of type 1 diabetes [2, 24–26]. At 4 weeks of age, the islets of NOD mice display a normal morphology and function but they are then progressively invaded by immune cells, resulting in beta cell dysfunction and death and, starting from weeks 12–14, in the appearance of diabetes [27]. To assess the potential contribution of tRFs to the initial phases of type 1 diabetes, we isolated the RNA from islets of NOD mice and treated the samples to remove the most common modifications [28]. We then compared, by small RNA-seq, the level of tRFs in the islets of 4 weeks old NOD mice (control) and of 8 weeks old NOD mice, which are still normoglycaemic but display insulitis [ESM Fig. 1] [29]) (Fig. 1). This led to the identification of ~20,000 tRFs, 158 of which were upregulated and 178 downregulated in prediabetic mice (*p*<0.05, fold change ≥2) (Fig. 1a, b and ESM Table 3; GEO accession no. GSE242568). Among the upregulated tRFs, 76 were generated from mitochondrial tRNAs (mt-tRFs, indicated by squares in Fig. 1b), while only ten downregulated tRFs originated from mitochondrial tRNAs (indicated by red squares in Fig. 1a). The modulation of several tRFs was confirmed by qPCR (Fig. 1c). The PCR primers were specifically designed for the sequences indicated in ESM Table 2 and ESM Fig. 2. Small RNA-seq of human islet samples (GEO accession no. GSE256343) permitted identification of close homologues of these tRFs (ESM Table 2).

### Proinflammatory cytokines modify the level of some tRNA fragments

Part of the changes in the tRF profile of prediabetic NOD mice may be triggered by exposure of islet cells to proinflammatory factors released by the invading immune cells. To determine the impact of proinflammatory cytokines, islet cells were treated with IL-1β, TNF-α and IFN-γ for 24 h, prior to tRF profiling. Prolonged exposure to these cytokines affected the level of 4974 tRFs (*p*<0.05, fold change ≥2), 179 of which displayed an FDR of <0.1. Among these, 50 were upregulated and 129 downregulated (ESM Table 4; GEO accession no. GSE242568).

**Fig. 2:**
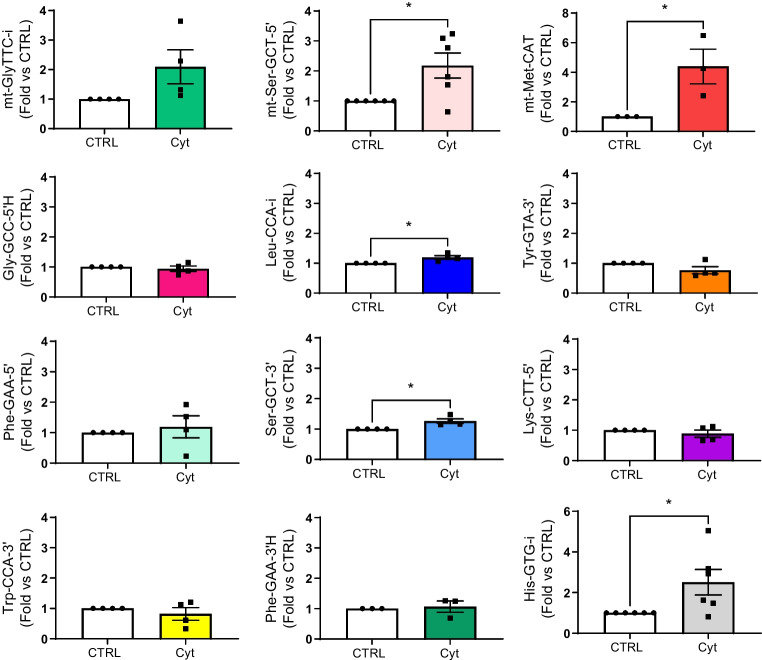
Identification of tRFs displaying changes in their levels in cytokine-treated islet cells. C57BL/6NRj mouse islet cells incubated with or without a mix of cytokines (IL-1β, IFN-γ and TNF-α) for 24 h before RNA collection. Real-time PCR validation of tRFs differentially expressed in cytokine-treated islet cells (Cyt) compared with control (CTRL). The data were normalised to the level of miR-7a and miR-375, which displays no changes in response to proinflammatory cytokines. The data are shown as fold changes vs CTRL and are presented as mean ± SD. *n*=3–6. *p<0.05 (paired *t* test). FC, fold change

As expected, part of the changes elicited by the cytokines overlapped with those observed in the islets of prediabetic NOD mice. Indeed, the level of mt-Met-CAT, mt-Ser-GCT-5′ and His-GTG-i were increased both in prediabetic NOD islet cells (Fig. 1c and ESM Table 3) and in response to cytokines (Fig. 2). However, at the time point investigated, many of the changes observed in prediabetic NOD mice were not reproduced by exposure to proinflammatory cytokines (Fig. 2). Treatment of human islets (see Human islet checklist in the ESM) with proinflammatory cytokines did not significantly affect the level of the measured tRFs (ESM Fig. 3).

IFN-α, a cytokine secreted by microbially infected cells, has been proposed to initiate innate immune responses and to contribute to type 1 diabetes development [30]. Thus, we analysed the impact of IFN-α on the selected mouse islet tRFs and found that only the level of mt-Met-CAT was significantly affected by this cytokine (ESM Fig. 4).

### tRNA fragments can be transferred from T lymphocytes to beta cells via extracellular vesicles

**Fig. 3:**
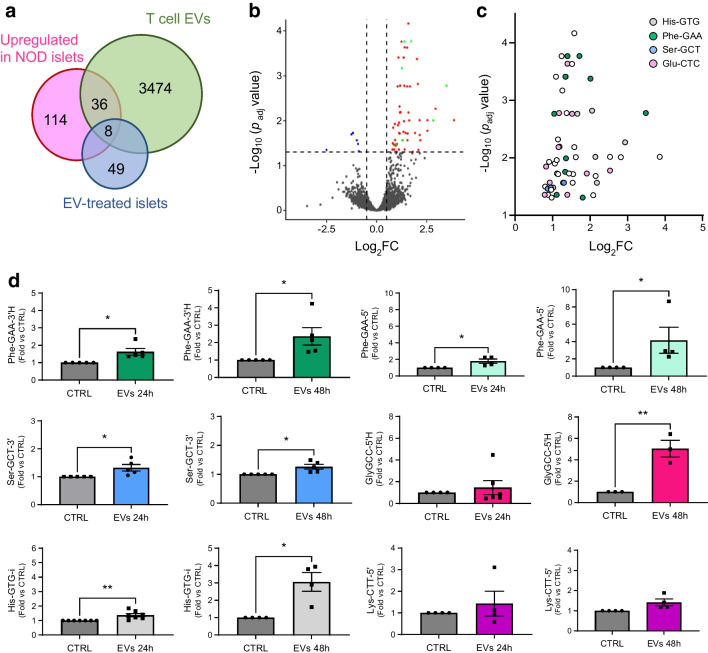
T cell EVs contain tRFs that are transferred to islet cells resulting in changes in the tRF pool. (**a**) Venn diagram showing the number of tRFs present in T cell EVs displaying changes in the islets of prediabetic NOD mice and in EV-treated islet cells. (**b**) Volcano plot indicating the changes in tRF level in islet cells after 24 h incubation with NOD mouse T cell EVs. Fragments above the horizontal dashed line display significant changes (*p*_*adj*_<0.05). Vertical dashed lines indicate a fold change of ±2. (**c**) Significantly upregulated tRFs in EV-treated mouse islet cells. Selected tRFs that originate from the same isodecoder tRNA are labelled with the same colour. (**d**) Analysis by qPCR of the level of selected tRFs in islet cells after 24 h and 48 h incubation with EVs released by NOD mouse T cells. The data were normalised to the level of miR-7a and miR-375, which display no changes under these experimental conditions; values are shown as fold change vs CTRL and are presented as mean ± SD. *n*=3 or 6. *p<0.05, **p<0.01 (ratio paired *t* test). FC, fold change

tRFs can be produced endogenously but can also be released and transferred in EVs to other cells [31, 32]. Since T cells are known to release EVs [33], we next investigated if the increased levels observed in prediabetic NOD islets could result from a transfer from T cell EVs to beta cells. EVs produced by activated CD4^+^/CD25^−^ T lymphocytes of 8-week-old female NOD mice displayed a mean diameter of ~150 nm and characteristic exosomal properties (ESM Fig. 5 and [4]). Small RNA profiling of these EV preparations (GEO accession no. GSE242568) revealed that, consistent with previous reports [34], tRFs represent 80% of the small non-coding RNA fraction (ESM Fig. 6a). Computational analysis identified 3518 unique tRF sequences in NOD T cell EVs (mean transcripts per million ≥0.5). Of note, most of the fragments present in the EVs originated from five isodecoder tRNAs (ESM Fig. 6b). Interestingly, many tRFs carried by EVs were upregulated in the islets of prediabetic NOD mice, pointing to a possible delivery of EV tRFs to beta cells (Fig. 3a and ESM Table 5).

To verify this hypothesis, islet cells were incubated in the presence of EVs released by NOD mouse T lymphocytes. RNA-seq analysis at the end of the treatment (GEO accession no. GSE242568) identified 57 tRFs that were increased in beta cells (*p*_*adj*_<0.05) upon exposure to EVs (Fig. 3a, b). Almost half of the increased tRFs originated from the fragmentation of three isodecoder tRNAs: Phe-GAA; Glu-CTC; and His-GTG (Fig. 3c and ESM Fig. 6c). The increase in several tRFs was confirmed by qPCR analysis of islet cells incubated for 24 h or 48 h with lymphocyte EVs (Fig. 3c). Similar changes were also observed in human islet cells incubated in the presence of EVs released by human CD4^+^ T cells (ESM Fig. 7) [4].

To investigate whether tRFs can be transferred from immune cells to beta cells, we developed an RNA-tagging approach. The tagging technique involved the incubation of donor cells with 5′-EU, a nucleotide derivative incorporated in cellular RNAs [35–37]. The tagged RNA released in EVs can be recovered after biotinylation of the EU residues and purification on streptavidin-coated beads (ESM Fig. 8a). To verify the efficiency of RNA tagging, Jurkat T cells and murine CD4^+^/CD25^−^ T lymphocytes were treated with EU for 24 h and 48 h, and the incorporation of the tagged nucleotide was confirmed by dot blot (ESM Fig. 8b). Quantitative PCR analysis of the RNA recovered on streptavidin beads revealed an efficient tagging of tRFs (ESM Fig. 8c). Moreover, the presence of tagged tRFs was also confirmed inside the EVs released by EU-treated T cells (ESM Fig. 8d).

**Fig. 4:**
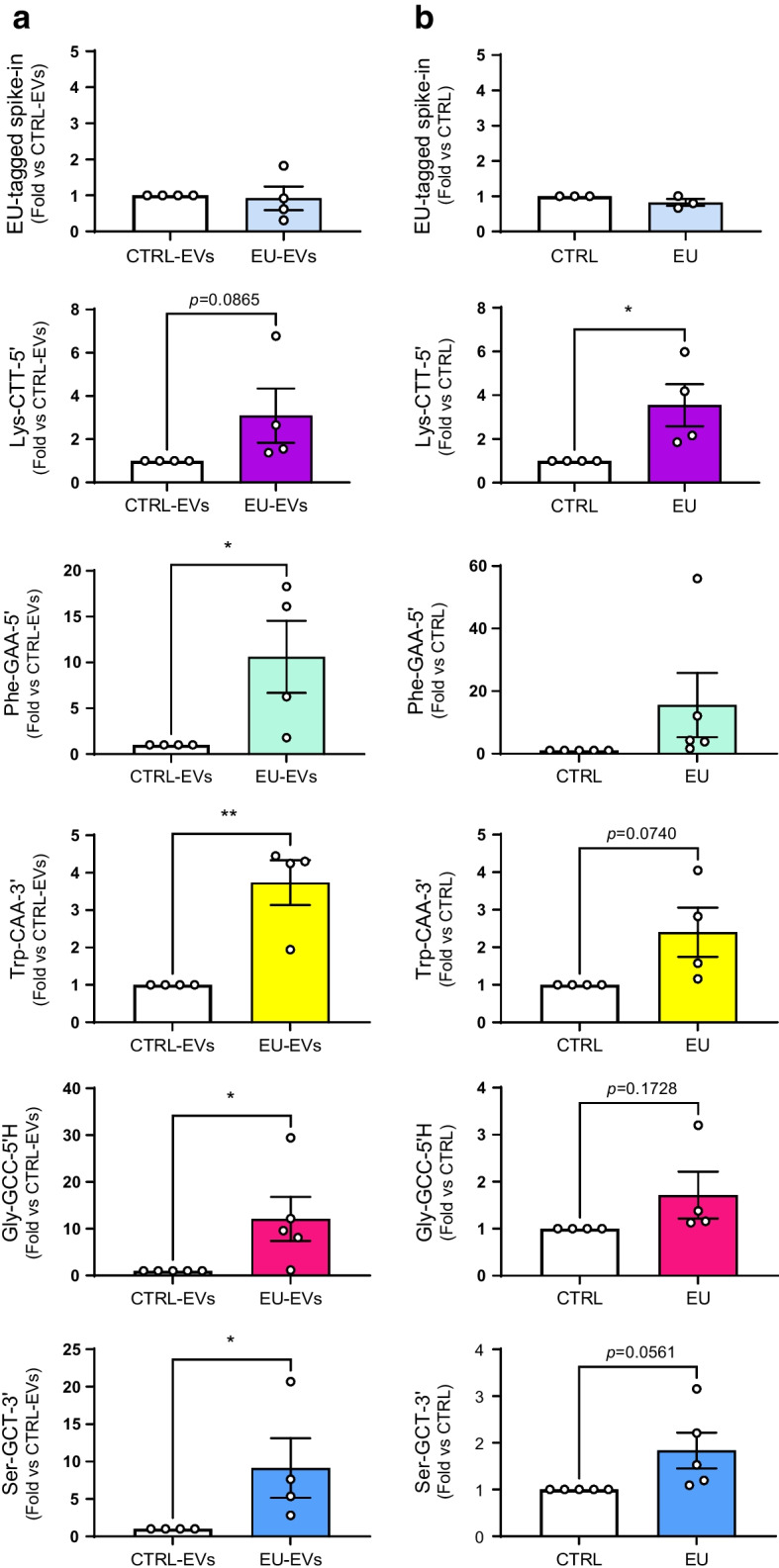
tRFs of CD4^+^/CD25^−^ T cells transferred in vitro and in vivo to pancreatic beta cells. (**a**) Real-time PCR of islet cell tRFs pulled down on streptavidin beads upon incubation with EVs released by EU-treated T cells (EU-EVs) or untreated control T cells (CTRL-EVs). An EU-tagged *C. elegans* miR-238 mimic was spiked in cell extracts as internal control. *p<0.05, **p<0.01 (ratio paired *t* test). (**b**) EU-tagged RNAs from FAC-sorted beta cells of NOD.SCID mice injected with EU-tagged T cells (EU) or with saline solution (CTRL) were purified on streptavidin beads and analysed by qPCR. An EU-tagged oligonucleotide containing the sequence of *C. elegans* miR-238 was spiked in the samples and was used as internal control to normalise the data. Data are presented as mean ± SD. *n*=3–5. *p<0.05 (ratio paired *t* test)

Next, islet cells were cultured in the presence of EVs produced by NOD mouse T lymphocytes previously incubated with (EU-EVs) or without (CTRL-EVs) EU. RNA was then extracted from islet cells, biotinylated and pulled down with streptavidin beads. An EU-tagged spike-in oligonucleotide containing the sequence of *C. elegans* miR-238 (ESM Fig. 8e) was used as internal control for the biotinylation and pull-down steps. The analysis of the pulled-down RNA revealed that islet cells treated with EU-EVs contained several tRFs produced by NOD mouse T lymphocytes (Fig. 4a). The tRF mt-Gln-TTG was used as negative control, since it was expressed in islet cells but not in T cell EVs (ESM Fig. 8f).

### Some tRNA fragments are transferred from T lymphocytes to beta cells during the early phases of type 1 diabetes

These in vitro findings were confirmed in vivo, using CD4^+^/CD25^−^ T lymphocytes from NOD BDC2.5 mice that, upon inoculation into NOD.SCID mice, promote the appearance of diabetes within less than 10 days [26, 38–41] (ESM Fig. 9a). CD4^+^/CD25^−^ T cells from NOD BDC2.5 mice were isolated and incubated with EU for 48 h. The cells were then inoculated into NOD.SCID mice by i.v. injection. We verified by dot blot that RNA molecules were still tagged 3 days after the removal of EU (ESM Fig. 9b) and that the injected T lymphocytes contained EU-tagged RNA (ESM Fig. 9c). Control mice were injected either with saline solution (SHAM) (CTRL), or with BDC2.5 mouse T cells not treated with EU (CTRL2). Two days after injection (the time taken for T cells to invade the islets [38]), animals were killed and beta cells sorted by FACS. At the time of islet collection, the animals were normoglycaemic (ESM Fig. 9d), suggesting that they were still in the initial phases of type 1 diabetes [26, 38–41]. The RNA isolated from purified beta cells was biotinylated and pulled down with streptavidin beads prior to RNA-seq profiling (ESM Fig. 9a). An EU-containing spike-in oligonucleotide was used as internal control for the biotinylation and the pull-down steps. As expected, the same amount of spike-in oligonucleotide was recovered in EU and CTRL samples (Fig. 4b). Bioinformatics analysis led to the identification of 141 unique tRF sequences with more reads in beta cells from NOD.SCID mice injected with EU-tagged T cells than in beta cells injected with saline solution (CTRL1) (ESM Table 5; GEO accession no. GSE242568). Similar results were obtained by comparing the RNAs pulled down from beta cells of NOD.SCID mice injected with EU-tagged T cells with those pulled down from beta cells of NOD.SCID mice injected with untagged T cells (CTRL2) (ESM Fig. 9e). The sequencing results for several tRFs were confirmed by qPCR (Fig. 4b). Interestingly, these tRFs were present in T cell EVs and were upregulated in islet cells of 8-week-old NOD mice (Fig. 1c and ESM Table 4) but not in islet cells of NOD.SCID mice (ESM Fig. 10), which do not develop diabetes. This confirms the hypothesis that the increased levels of these tRFs in islet cells under prediabetic conditions is caused by the transfer from the invading T cells rather than by the exposure of beta cells to proinflammatory cytokines.

### Impact of transferred tRNA fragments on beta cell function

Five tRF candidates were selected for functional studies based on the following criteria: (1) their increase in NOD islet cells during insulitis; (2) their increase in beta cells after exposure to T cell EVs; and (3) their transfer from T cells to beta cells in vitro and/or in vivo, in a model of autoimmune diabetes.

**Fig. 5:**
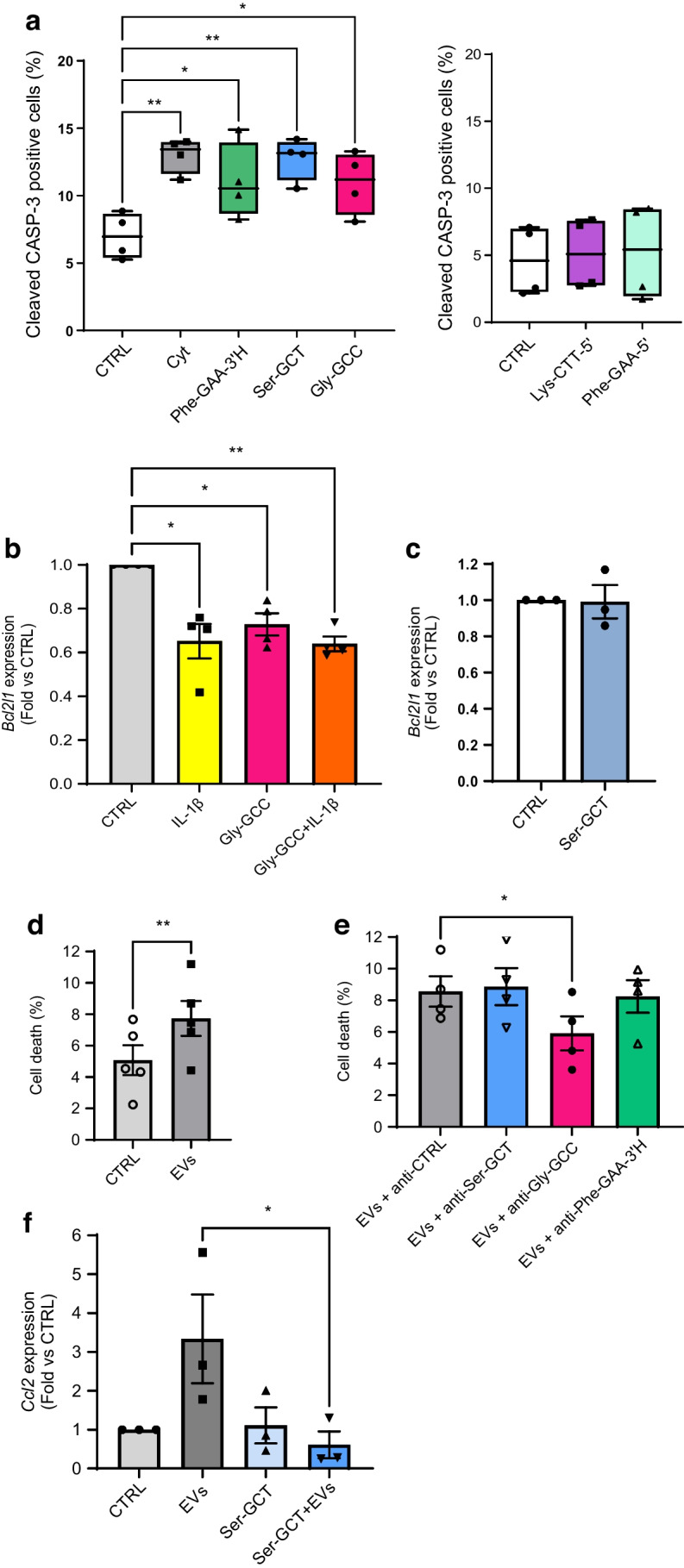
Modulation of the levels of selected tRFs affects beta cell apoptosis. (**a**) Mouse islet cells were transfected with different tRF mimics (as indicated) or with a scrambled control sequence (CTRL) for 48 h. Part of the cells were incubated for 24 h with a mix of proinflammatory cytokines (IL-1β, IFN-γ and TNF-α) (Cyt) prior to staining using antibodies against insulin and cleaved caspase-3 (CASP-3). Between 600 and 1000 cells per condition were counted and the percentage of cleaved caspase-3 positive beta cells was calculated. *p<0.05, **p<0.01 (one-way ANOVA), *n*=4 (Dunnett Correction). (**b**) MIN6 cells were transfected with an oligonucleotide containing the sequence of Gly-GCC-5′H (Gly-GCC) or with a scrambled sequence (CTRL) for 48 h and were subsequently incubated with or without IL-1β for 24 h. RNA was collected and *Bcl2l1* expression was measured by qPCR. *p<0.05, **p<0.01 (one-way ANOVA), *n*=4 (Dunnett correction). (**c**) MIN6 cells were transfected with an oligonucleotide containing the sequence of Ser-GCT or with a scrambled sequence (CTRL). Two days later, RNA was collected and *Bcl2l1* expression was measured by qPCR. (**d**, **e**) Mouse pancreatic islet cells were transfected with a control oligonucleotide inhibitor (CTRL) or with a tRF inhibitor as indicated. After 24 h, the cells were treated with T cell EVs and incubated for another 48 h. Islet cell death was assessed by scoring the cells displaying pycnotic nuclei upon Hoechst/propidium iodide staining (around 5000 cells were counted per condition). **p<0.01 (paired *t* test), *n*=5 (**d**); *p<0.05 (one-way ANOVA, Dunnett post hoc test), *n*=4 (**e**). (**f**) MIN6 cells were transfected with Ser-GCT-3′ inhibitor or a negative control (CTRL). After 24 h, the cells were incubated with or without T cell EVs for another 24 h. RNA was collected and *Ccl2* expression was measured by qPCR. *p<0.05 (one-way ANOVA, Šidák correction), *n*=3. (**a**) Data are presented as median, with 25th and 75th percentile, and whiskers showing minimum and maximum. (**b**–**f**) Data are presented as mean ± SD

Exposure to NOD T cell EVs induces beta cell death [4]. We investigated whether this effect was at least in part mediated by an increase in tRFs. For this purpose, the levels of candidate tRFs were increased by transfecting mouse islet cells with oligonucleotide mimics (ESM Fig. 11a) and by assessing beta cell death. Propidium iodide / Hoechst staining and immunofluorescence analysis with anti-insulin and anti-cleaved caspase-3 antibodies showed a rise in beta cell apoptosis when the levels of Phe-GAA-3ʹH, Ser-GCT-3′ or Gly-GCC-5ʹH were increased (Fig. 5a and ESM Fig. 11b). In contrast, overexpression of Lys-CTT-5′ and Phe-GAA-5′ did not affect beta cell survival (Fig. 5a). The effect of Gly-GCC-5’H on beta cell apoptosis could be partially explained by the inhibition of the anti-apoptotic protein Bcl-XL. Indeed, the overexpression of Gly-GCC-5ʹH triggered a 40% decrease in *Bcl2l1* mRNA levels (Fig. 5b). This effect was specific, since overexpression of Ser-GCT-3′ had no effect on the expression of this anti-apoptotic gene (Fig. 5c).

As expected, incubation of mouse islet cells with EVs released by NOD mouse T cells led to an increase in cell death (Fig. 5d). While inhibition of Ser-GCT-3′, Gly-GCC-5ʹH or Phe-GAA-3ʹH did not affect apoptosis under basal conditions (ESM Fig. 11d), the introduction of Gly-GCC-5ʹH antisense oligonucleotides into islet cells 24 h before exposure to T cell EVs prevented the increase in tRF levels (ESM Fig. 11c) and reduced cell death induced by EVs (Fig. 5e). Inhibition of Ser-GCT-3′ and Phe-GAA-3′H (ESM Fig. 11c) in the receiving beta cells was not sufficient to prevent apoptosis triggered by EVs (Fig. 5e). However, blockade of Ser-GCT-3′ was found to prevent the rise in *Ccl2* expression levels induced by EV (Fig. 5f), suggesting the possible involvement of this tRF in the recruitment of immune cells.

**Fig. 6:**
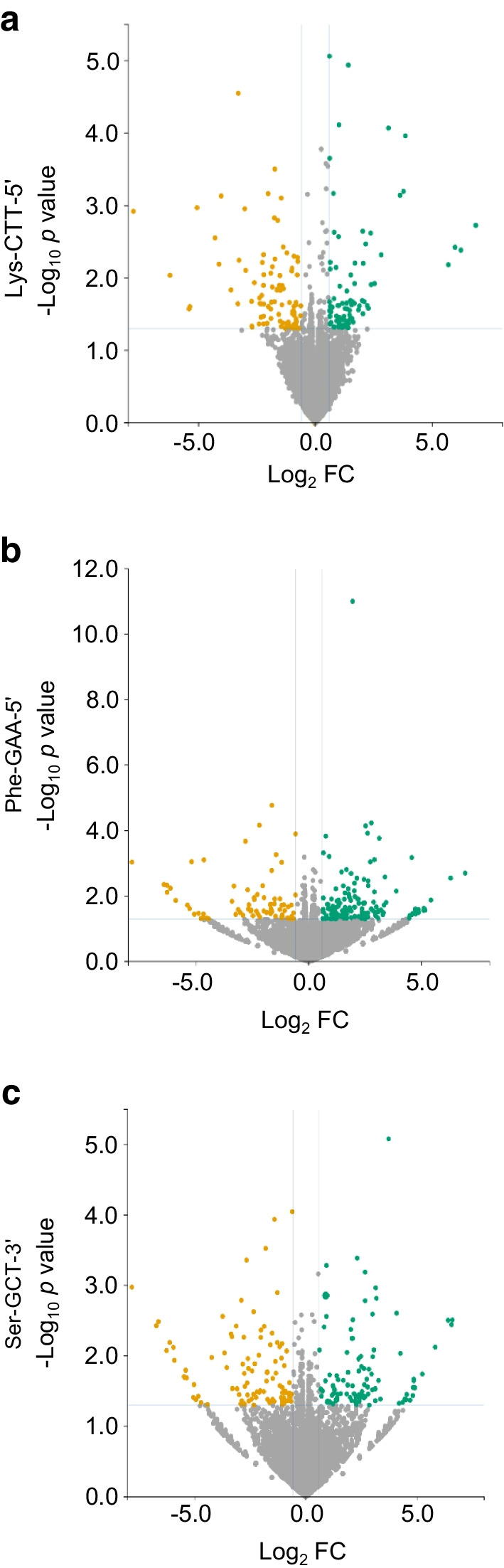
Overexpression of Lys-CTT-5′, Phe-GAA-5′ or Ser-GCT-3′ affects islet cell gene expression. Volcano plots of the differentially expressed transcripts in Lys-CTT-5′ (**a**), Phe-GAA-5′ (**b**) or Ser-GCT-3′ (**c**) overexpressing islet cells compared with cells transfected with a scrambled oligonucleotide. Upregulated transcripts are shown in green and downregulated transcripts are shown in orange. FC, fold change

**Fig. 7:**
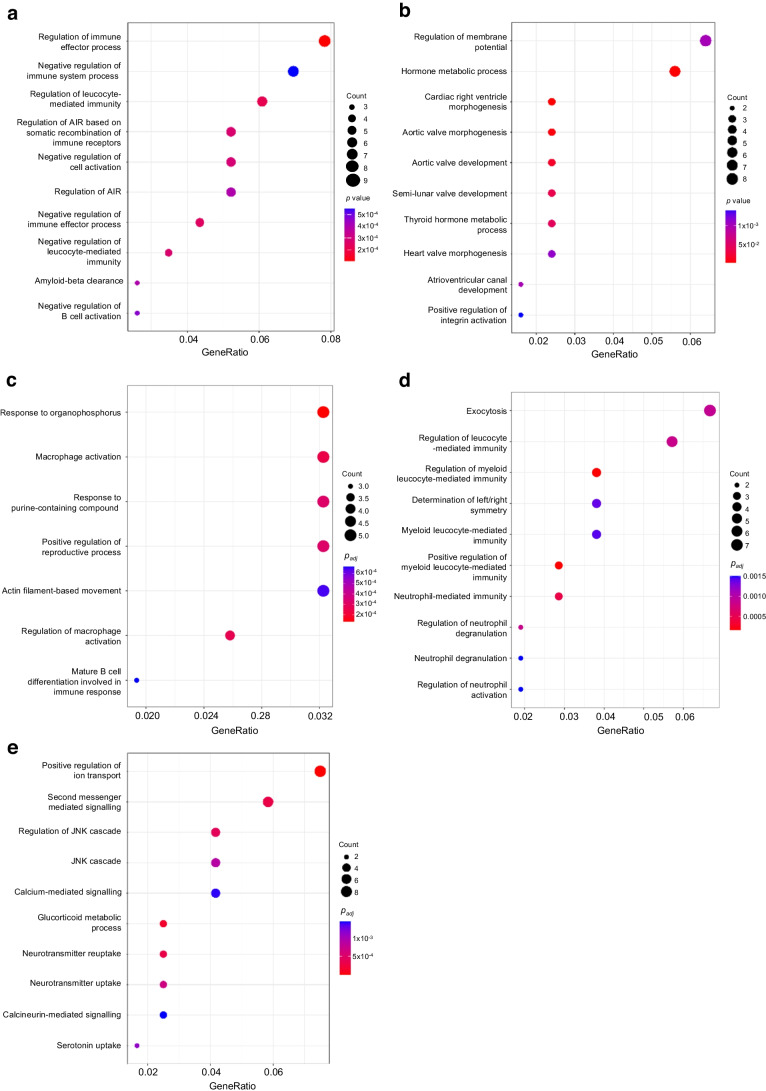
GO analysis of the transcripts affected by the overexpression of Lys-CTT-5′, Phe-GAA-5′ or Ser-GCT-3′. (**a**, **b**) Top enriched GO terms for downregulated (**a**) and upregulated (**b**) genes in mouse islet cells overexpressing Lys-CTT-5′. (**c**, **d**) Top enriched GO terms for downregulated (**c**) and upregulated genes (**d**) in mouse islet cells overexpressing Phe-GAA-5′. (**e**) Top enriched GO terms for upregulated genes in mouse islet cells overexpressing Ser-GCT-3′. The size of the circles is proportional to the ratio of the observed vs the expected overlaps. *p* values correspond to hypergeometric tests (colour-coded)

To gain insight into the role of the tRFs transferred in beta cells, we performed bulk RNA-seq analysis in mouse islet cells overexpressing Lys-CTT-5′, Phe-GAA-5′ or Ser-GCT-3′ (GEO accession no. GSE242568). After 48 h overexpression of Lys-CTT-5′, 167 genes were upregulated (fold change >1.5, *p*<0.05) and 151 were downregulated (fold change >−1.5, *p*<0.05) (Fig. 6a and ESM Table 7). Gene ontology (GO) analysis of the downregulated genes revealed an enrichment for transcripts associated with immune functions (Fig. 7a), including those regulating immune effector processes and leucocyte-mediated immunity. In contrast, the upregulated genes were mainly related to hormone metabolic processes (Fig. 7b).

Interestingly, GO analysis revealed that the upregulation of Phe-GAA-5′ also affects genes involved in the activation of the immune system. Indeed, many of the 334 genes differentially expressed in Phe-GAA-5′ transfected cells (Fig. 7c, d and ESM Table 8) are associated with inflammation and immune functions, such as the regulation of TNF production.

The analysis of mouse islet cells overexpressing Ser-GCT-3′ led to the identification of 152 upregulated and 158 downregulated genes (fold change >1.5, *p*<0.05) (Fig. 7e and ESM Table 9). GO analysis of the upregulated transcripts highlighted an enrichment for genes regulating Jun N-terminal kinase activity. Since the activation of this kinase is known to induce beta cell death [42, 43], this may contribute to the apoptosis observed after Ser-GCT-3′ overexpression.

Taken together, these observations indicate that the transfer of tRFs from CD4^+/^CD25^−^ T lymphocytes to beta cells during insulitis modify the transcriptomic landscape of the insulin-secreting cells, making them more engaged in the regulation of the immune cells and rendering them more susceptible to the autoimmune reaction.

## Discussion

During the initial phases of type 1 diabetes, immune cells invade the islets of Langerhans and promote beta cell dysfunction and apoptosis. We and others have shown that exposure of the insulin-secreting cells to proinflammatory cytokines released by the invading immune cells leads to changes in the level of different classes of non-coding RNAs [29, 44–46]. Moreover, a pool of microRNAs is directly transferred to beta cells via EVs and their blockade in beta cells prevents the development of type 1 diabetes in NOD mice [4]. tRFs are very abundant in EVs [34] but their potential transfer from T cells to beta cells has never been explored. Previous data from our laboratory indicate that tRFs are involved in beta cell mass expansion and in the functional maturation of neonatal beta cells [18]. Moreover, inappropriate levels of some tRFs have been shown to trigger beta cell apoptosis [10]. In this study, we provide a comprehensive view of the modulation of the tRF pool in beta cells during autoimmunity and highlight the potential contribution made by these non-coding RNAs to beta cell dysfunction and loss.

Using NOD mice as a model of type 1 diabetes, we demonstrate that the tRF profile of islet cells is altered during the initial phases of the disease. The changes in the tRF pool result from the crosstalk with the immune cells entering the islets. We found that proinflammatory cytokines released by the immune cells induce the upregulation of many tRFs produced by mitochondrial tRNA fragmentation. Exposure of beta cells to cytokines has been shown to induce mitochondrial dysfunction [47]. Thus, dysregulation of the mitochondrial tRF pool may contribute to these deleterious effects of the cytokines.

Using an RNA-tagging approach we could show that a fraction of the tRFs displaying elevated levels during insulitis is directly transferred from autoreactive T cells to beta cells via EVs. Oxidative stress associated with T cell activation triggers tRNA fragmentation and the release of the generated tRFs in EVs [34]. Our data suggest that at least some of these tRFs are transferred to target cells and may participate in the immune reaction. Indeed, the EVs released by NOD mouse CD4^+^/CD25^−^ T cells modify the tRF pool of beta cells and trigger apoptosis. These results suggest that the transfer of T cell tRFs during the initial phases of autoimmune diabetes participates in beta cell demise and to the development of type 1 diabetes. Data obtained with human islet preparations confirm the relevance of our findings for human pathology.

A transfer of genetic material has been hypothesised in many physiological and pathological conditions but only a few studies have provided experimental evidence for this phenomenon. An RNA-tagging technique has been used to demonstrate the transfer of RNAs from epididymis to sperm [48]. However, in that study the authors were unable to identify specific RNA molecules shuttling between the cells in vivo. Our EU-tagging technique led to the identification of several tRFs delivered to beta cells upon adoptive transfer of activated CD4^+^/CD25^−^ T cells from NOD.BDC 2.5 mice, a treatment known to trigger type 1 diabetes in the receiving NOD.SCID mice within a few days [38, 39, 41]. These findings provide a proof of principle of the transfer of tRFs from T lymphocytes to beta cells during the initial phases of the disease.

In this study, we investigated the function of tRFs delivered to beta cells during insulitis. The overexpression of these fragments results in beta cell apoptosis or affects the level of genes involved in the activation of the immune system. We observed an increase in beta cell apoptosis upon overexpression of Gly-GCC-5′H and blockade of this tRF protected beta cells from EV-induced apoptosis. These findings are in agreement with observations in other cells [49, 50]. In beta cells, the effect of Gly-GCC-5′H is probably mediated by diminished *Bcl2l1* expression. Indeed, reduction of B-cell lymphoma-extra large (Bcl-XL) expression is a key event in beta cell apoptosis [51] and higher expression levels of this protein in alpha cells was suggested to protect glucagon-secreting cells against cytokine-induced cell death [52, 53].

Overexpression of Ser-GCT-3′ has a similar effect on beta cell survival but the mechanism appears to be independent from changes in Bcl-XL level. Transcriptomic analysis of Ser-GCT-3′-overexpressing cells revealed an enrichment of genes involved in JNK activation, potentially explaining the induction of apoptosis. Indeed, cytokine- and palmitate-dependent JNK activation elicits beta cell apoptosis [42, 43].

Overexpression of Lys-CTT-5′ and Phe-GAA-5′ did not affect beta cell survival but interfered with the expression of genes involved in immune cell activation. Indeed, higher levels of Lys-CTT-5′ and Phe-GAA-5′ were associated with the downregulation of transcripts linked to the control of the immune function. Thus, although these tRFs do not directly impact beta cell survival, they may sensitise insulin-secreting cells to the autoimmune reaction.

Our data demonstrate that the crosstalk with CD4^+^/CD25^−^ T cells during the initial phases of type 1 diabetes triggers changes in the tRF beta cell landscape that contributes to the demise of the insulin-secreting cells. tRFs can mediate their action through a variety of different mechanisms by interacting with proteins and/or other RNAs [6]. The precise mechanisms by which the tRFs displaying changes under diabetes conditions affect beta cell function remain to be elucidated and will need to be investigated in future studies.

Our findings extend the repertoire of non-coding RNAs involved in the control of beta cell function and diabetes pathogenesis. tRFs are abundant in EVs. Thus, these molecules may potentially constitute novel biomarkers to predict the development of type 1 diabetes. Our observations have implications going beyond the diabetes field. In fact, the transfer of genetic material between T cells and their targets is likely to occur in association with other autoimmune reactions and to be relevant for the understanding of the pathogenesis of other human diseases.

## Supplementary Information

Below is the link to the electronic supplementary material.

Supplementary file1 (PDF 1441 KB)

Supplementary file2 (XLSX 5959 KB)

## Data Availability

Sequence data are available from the Gene Expression Omnibus (GEO) (https://www.ncbi.nlm.nih.gov/geo/) with accession numbers GSE242568 and GSE256343.

## Abbreviations

EU: Ethynyl uridine
EV: Extracellular vesicle
FDR: False discovery rate
GEO: Gene expression omnibus
GO: Gene ontology
qPCR: Quantitative PCR
tRF: tRNA-derived fragment
