# Supplementary material for: tRNA-derived fragments in T lymphocyte–beta cell crosstalk and in type 1 diabetes pathogenesis in NOD mice
Source: Diabetologia. 2024 Jul 5;67(10):2260–74. doi: 10.1007/s00125-024-06207-3 (PMC11446995; doi:10.1007/s00125-024-06207-3)
Supplement: Supplementary file 1 — Supplementary file1 (PDF 1441 KB) [file 125_2024_6207_MOESM1_ESM.pdf]

SUPPLEMENTARY METHODS

tRF annotation and classification

tRFs were classified by their positions in the mature tRNA. We defined five structural categories comprising: (a) “5′-tRNA halves” (5′-tRHs) beginning at position +1 (or, −1) of the tRNA and terminating at any of the four underlined positions n1 ∇n2 ∇A1 ∇A2 ∇A3 n3 n4 (each ∇ denotes a reported Angiogenin cleavage site at the anticodon); (b) “3′-tRNA halves” (3′-tRHs) originating from any of the four underlined positions n1 ∇n2 ∇A1 ∇A2 ∇A3 n3 n4 and terminating in the first C, the second C, or the A of the non-templated CCA addition. (c) “5′-tRFs” beginning at the 5′ end (+1 or −1) of the tRNA and ending in a position other than n1, n2, A1, or A2. (d) “i-tRFs” (internal tRFs) originating from either position +2 or further to the right and end before the first C of the non-templated CCA addition. (e) “3′-tRFs” beginning at a position other than n2, A1, A2 or A3 and terminating at the first C, the second C, or the A of the non-templated CCA addition.

RNAseq analysis of pulled-down EU-tagged RNA

The RNA reads analysed have the following structure (from 5'-end to 3'end):

- Captured RNA molecule
- 3' Adapter AACTGTAGGCACCATCAAT
- UMI of 12 nucleotides
- Primer site AGATCGGAAGAGCACACGTCTGAACTCCAGTCAC
- barcode of 6 nucleotides
- P7 capture primer ATCTCGTATGCCGTCTTCTGCTTGA
- poly-A

We wrote a Perl script to analyze those reads, which first locates the 3' Adapter and then extracts the captured RNA molecule sequence (the leading part of the read up to the adapter). The script then locates the Primer site and extracts the 12 nucleotides UMI. Next the script extracts the 6 nucleotide barcode and compares it to the expected barcode(s) for the sample being analyzed. If the 3' Adapter, the Primer site, or the barcode are not recognized, the read is ignored. If the sites and barcode are correctly recognized, the sequence is kept to record the number of different UMIs observed. The final output of the script is a tab-delimited text file listing the count of distinct sequences for each sample. We obtained 13’323’134 sequences, which were filtered to retain only sequences of 16-55 nucleotides length (11’984’754 distinct sequences). These were further filtered to keep only sequences with at least 5 counts in at least 4 samples (63367 distinct sequences). Finally, we retained only sequences that could originate from fragments of the 400 "high confidence" and 7 "notable atypical predictions" tRNA

sequences retrieved from http://gtrnadb.ucsc.edu/genomes/eukaryota/Mmusc39/ or be fragments of the control miR-238 C. elegans sequence spiked in each sample. The 253 remaining sequences were analyzed with R (version 4.2.2) and DEseq2 (version 1.38.3; [1]) with default parameters.

Annotated results are provided in ESM Table 5, with samples raw counts, DEseq2 normalized counts, and rlog counts after applying a variance stabilizing transformation (VST) using the vst function taking in to account the experimental design (blind=FALSE). Foldchange computed by DEseq2 and moderated by the lfcShrink function using the apeglm [2] shrinkage estimator are also provided in columns Fold Change and Fold Change_apeglm, respectively.

References

1. Love, M.I., W. Huber, and S. Anders, *Moderated estimation of fold change and dispersion for RNA-seq data with DESeq2.* Genome Biol, 2014. **15**(12): p. 550.
2. Zhu, A., J.G. Ibrahim, and M.I. Love, *Heavy-tailed prior distributions for sequence count data: removing the noise and preserving large differences.* Bioinformatics, 2019. **35**(12): p. 2084-2092.

ESM Table 1

Key resource table

REAGENT/RESOURCE	REFERENCE OR SOURCE	IDENTIFIER OR CATALOG NUMBER
**ANTIBODIES**		
APC anti-CD3	BioLegend	#100235
Brilliant Violet 421 anti-CD4	BioLegend	#100437
FITC anti-CD45	BioLegend	#157213
PE anti-CD25	BioLegend	#100007
TruStain FcX™ (anti-mouse CD16/32) antibody	BioLegend	#101319
Goat anti-guinea pig IgG Alexa Fluor 488	Thermo Fisher Scientific	#A-11073
Goat anti-rabbit IgG Alexa Fluor 555	Thermo Fisher Scientific	#A-21428
Guinea pig anti-insulin, polyclonal	Agilent	IR00261-2
Rabbit anti-caspase-3 (Asp 175)	Cell Signaling	#9661
**CHEMICALS, PEPTIDES AND ENZYMES**		
ß-Mercaptoethanol	Merck-Sigma	#M6250
Bovine serum albumin BSA	Merck-Sigma	#6003
CD4+ CD25+ Regulatory T Cell Isolation Kit, mouse	Miltenyi Biotec GmbH	# 130-091-041
Click-iT Nascent RNA Capture kit	Thermo Fischer Scientific	#C10365
Collagenase	Merck-Sigma	#C9263 for mouse islets
DNase	Promega	#M6101
Dulbecco's modified Eagle's medium (DMEM)	Thermo Fisher Scientific	#41966
Dynabeads Mouse T-Activator CD3/CD28 beads	Thermo Fisher Scientific	# 11453D
5-ethynyl uridine (EU)	Baseclick	#BCN-003

Fetal bovine serum	Thermo Fisher Scientific	#2720801
	Merck-Sigma	#F7524
Fluor-Save mounting medium	Merck-Sigma	#345789
Hepes	Merck-Sigma	#H3375
Histopaque density gradient	Merck-Sigma	#1077 and #1119
Hoechst 33342	Thermo Fisher Scientific	#H3570
Interleukin-1β (IL-1β)	PeproTech	#211-11B
Interleukin-2, recombinant mouse (IL-2)	PeproTech	#212-12
Interleukin-12, recombinant mouse (IL-12)	PeproTech	#210-12
Interferon-γ (IFN-γ)	PeproTech	#315-05
Interferon-α (IFN-α)	Abcam	#ab259386
Lipofectamine 2000	Thermo Fischer Scientific	#11668019
miRCURY LNA Universal RT microRNA PCR system	Qiagen	#339340 for RT kit and #339347 for Sybr Green
M-MLV reverse transcriptase (Promega #M3683)	Promega	#M3683
miRNeasy micro kit	Qiagen	#217084
miRNeasy mini kit	Qiagen	#217004
Penicillin streptomycin	Thermo Fisher Scientific	#15140122
Pyruvate	Merck-Sigma	#P2256
QIAseq miRNA NGS 48 Index IL kit	Qiagen	#331595
QIAzol lysis reagent	Qiagen	#79306
Random primers	Promega	#C1181
Roswell Park Memorial Institute (RPMI) 1640 GlutaMAX medium	Thermo Fischer Scientific	#72400-054
rtStar™ tRF&tiRNA Pretreatment kit	Arraystar	#AS-FS-005
Sodium pyruvate	Merck-Sigma	#S-8636
Streptavidin dynabeads	Thermo Fischer Scientific	#M-280 Streptavidin 11206D

SsoAdvanced Universal SYBR Green Supermix	BioRad	#1725274
Sterile saline solution	Merck-Sigma	#C5914
Trypsin	Thermo Fischer Scientific	#15400-054
Tumor Necrosis Factor-α (TNF-α)	PeproTech	#315-01A
**EXPERIMENTAL MODELS**		
Male C57BL/6NRj (aged 12 weeks)<br>(*M. musculus*)	Janvier Laboratories	Non applicable
Female NOD.CB-17-Prkdc scid/Rj (aged 4 and 8 weeks)<br>(*M. musculus*)	Charles River	Non applicable
Female NOD.Cg-Tg (TcraBDC2.5, TcrbBDC2.5) 1Doi/DoiJ, HEM (aged 8 weeks)<br>(*M. musculus*)	Jackson Laboratory	Non applicable
MIN6B1 (*M. musculus*)	Janvier Laboratories	Non applicable
**SOFTWARES AND INSTRUMENTS**		
AxioCam MRc5	Zeiss	Non applicable
Axiovision fluorescence microscope	Zeiss	Non applicable
QIAGEN RNA-seq Analysis Portal 4.1	Qiagen	Non applicable
Zetasizer Ultra	Malvern Panalytical	Non applicable

**Primers used with SsoAdvanced Universal SYBR Green Supermix (Biorad)**			
Species	Targets	Forward primers (5'-3')	Reverse primers (5'-3')
Mus Musculus	*Bcl2l1*	TAAACTGGGGTCGCATTGTG	AGGTAAGTGGCCATCCAAGC
	Ccl-2	AGGTGTCCCAAAGAAGCTGT	ACAGAAGTGCTTGAGGTGGT

Diabetologia

Checklist for reporting human islet preparations used in research

Adapted from Hart NJ, Powers AC (2018) Progress, challenges, and suggestions for using human islets to understand islet biology and human diabetes. Diabetologia https://doi.org/10.1007/s00125-018-4772-2

Islet preparation	1	2	3	4	5	6	7	8[a]
**MANDATORY INFORMATION**								
Unique identifier	HI9	HI12	HI7	N/A	N/A	H1046	HP-18157-01	HI14
Donor age (years)	59	60	54	53	39	50	43	47
Donor sex (M/F)	F	F	M	M	F	M	F	F
Donor BMI (kg/m^2)	23.7	22.2	28.5	27.2	31.4	19.6	34.0	33.4
Donor HbA_{1c} or other measure of blood glucose control	-	-	-	-	-	-	4.6	-
Origin/source of islets[b]	ECIT	ECIT	ECIT	CEED	CEED	ECIT	Tet-Bio	ECIT
Islet isolation centre	Geneva	Geneva	Geneva	Strasbourg	Strasbourg	Geneva		Geneva
Donor history of diabetes? Please select yes/no from drop down list	No	No	No	No	No	No	No	No
**If Yes, complete the next two lines if this information is available**								
Diabetes duration (years)								
Glucose-lowering therapy at time of death[c]								
**RECOMMENDED INFORMATION**								
Donor cause of death	Cerebral trauma	Cerebral trauma	Cerebral trauma			Stroke	Stroke	Cerebral trauma
Warm ischaemia time (h)								
Cold ischaemia time (h)	7h39	7h30	3h20			5h30		3h20

Estimated purity (%)	84	94	60	80	70	80	90	88
Estimated viability (%)	90	90	89	90	80	95	95	90
Total culture time (h)[d]	9 days	8 days	8 days	6 days	6 days	7 days	8 days	6 days
Glucose-stimulated insulin secretion or other functional measurement[e]								
Handpicked to purity? Please select yes/no from drop down list								
Additional notes								

[a]If you have used more than eight islet preparations, please complete additional forms as necessary
[b]For example, IIDP, ECIT, Alberta IsletCore
[c]Please specify the therapy/therapies
[d]Time of islet culture at the isolation centre, during shipment and at the receiving laboratory
[e]Please specify the test and the results

Checklist for reporting human islet preparations used in research

Adapted from Hart NJ, Powers AC (2018) Progress, challenges, and suggestions for using human islets to understand islet biology and human diabetes. Diabetologia https://doi.org/10.1007/s00125-018-4772-2

**Islet preparation**	**1**	**2**	**3**	**4**	**5**	**6**	**7**	**8[a]**
**MANDATORY INFORMATION**								
Unique identifier	P656	P637	P612					
Donor age (years)	60	53	66					
Donor sex (M/F)	M	M	F					
Donor BMI (kg/m^2)	30.2	23.5	19.5					
Donor HbA_{1c} or other measure of blood glucose control								
Origin/source of islets[b]	ECIT	ECIT	ECIT					
Islet isolation centre	Geneva	Geneva	Geneva					
Donor history of diabetes? Please select yes/no from drop down list	No	No	No					
**If Yes, complete the next two lines if this information is available**								
Diabetes duration (years)								
Glucose-lowering therapy at time of death[c]								
**RECOMMENDED INFORMATION**								
Donor cause of death								
Warm ischaemia time (h)								
Cold ischaemia time (h)								

Estimated purity (%)								
Estimated viability (%)								
Total culture time (h)[d]								
Glucose-stimulated insulin secretion or other functional measurement[e]								
Handpicked to purity? Please select yes/no from drop down list								
Additional notes								

[a]If you have used more than eight islet preparations, please complete additional forms as necessary
[b]For example, IIDP, ECIT, Alberta IsletCore
[c]Please specify the therapy/therapies
[d]Time of islet culture at the isolation centre, during shipment and at the receiving laboratory
[e]Please specify the test and the results

NOD mice 4 weeks NOD mice 8 weeks

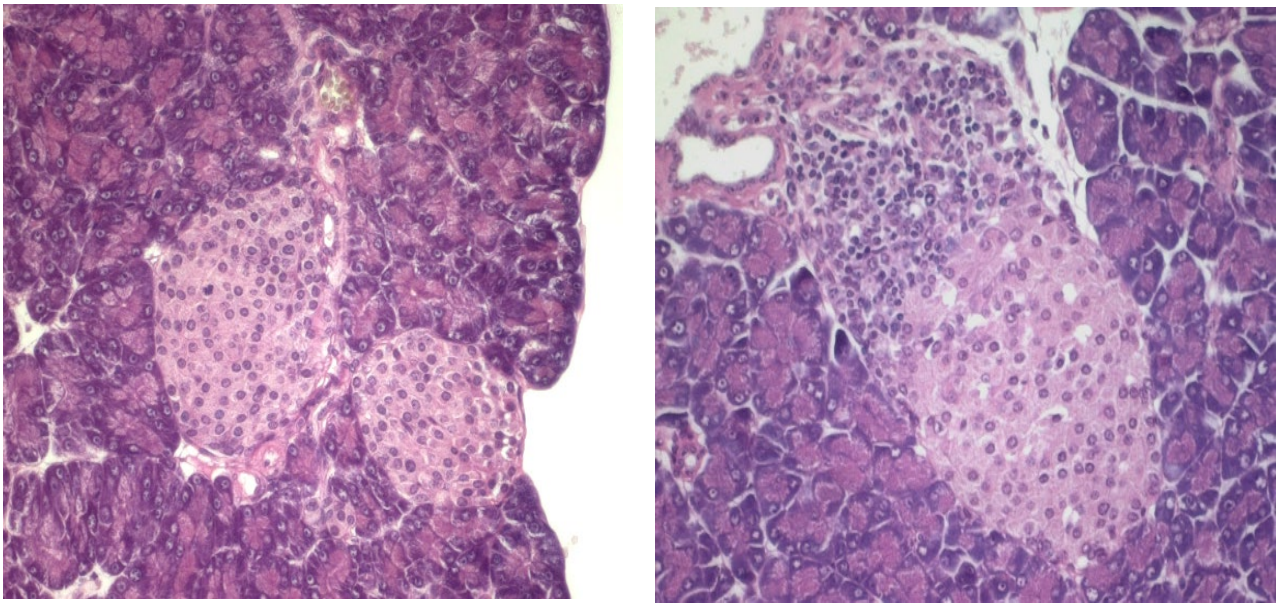

ESM Figure 1: Pancreatic insulitis in prediabetic NOD mice. Pancreatic slices of 4 and 8 week–old female NOD mice were stained by hematoxylin-eosin.

mt-Gly-TCC-i

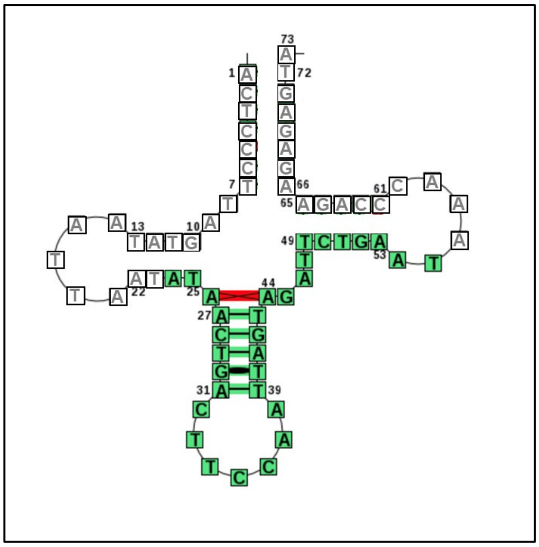

mt-Ser-GCT-5'H

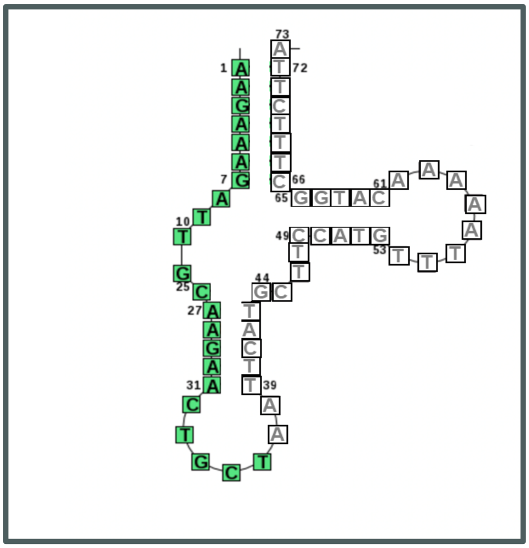

mt-Met-CAT-i

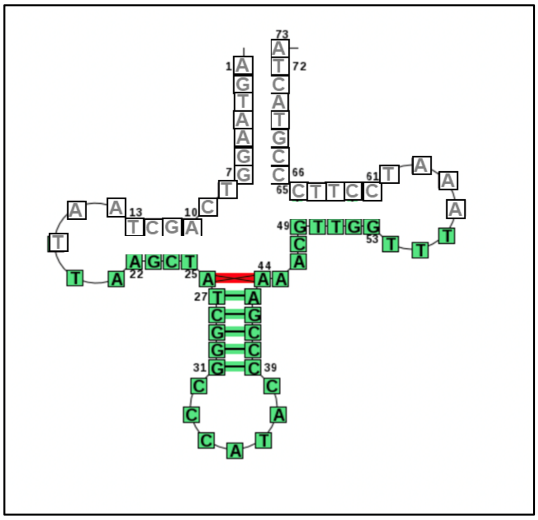

mt-Gln-TTG-5'H

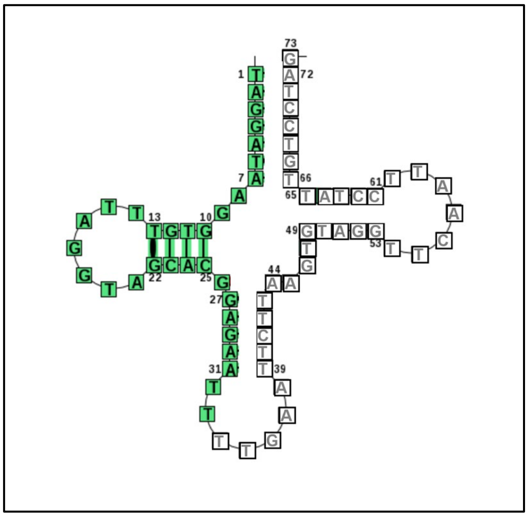

ESM Figure 2A: Selected mitochondrial tRNA-derived fragments displaying changes in NOD islets during insulitis. The selected tRFs are aligned to the secondary structure of the tRNAs of origin. The indicated tRFs represent the sequences amplified by qPCR in Fig. 1C.
Adapted from : http://mttrna.bioinf.uni-leipzig.de/

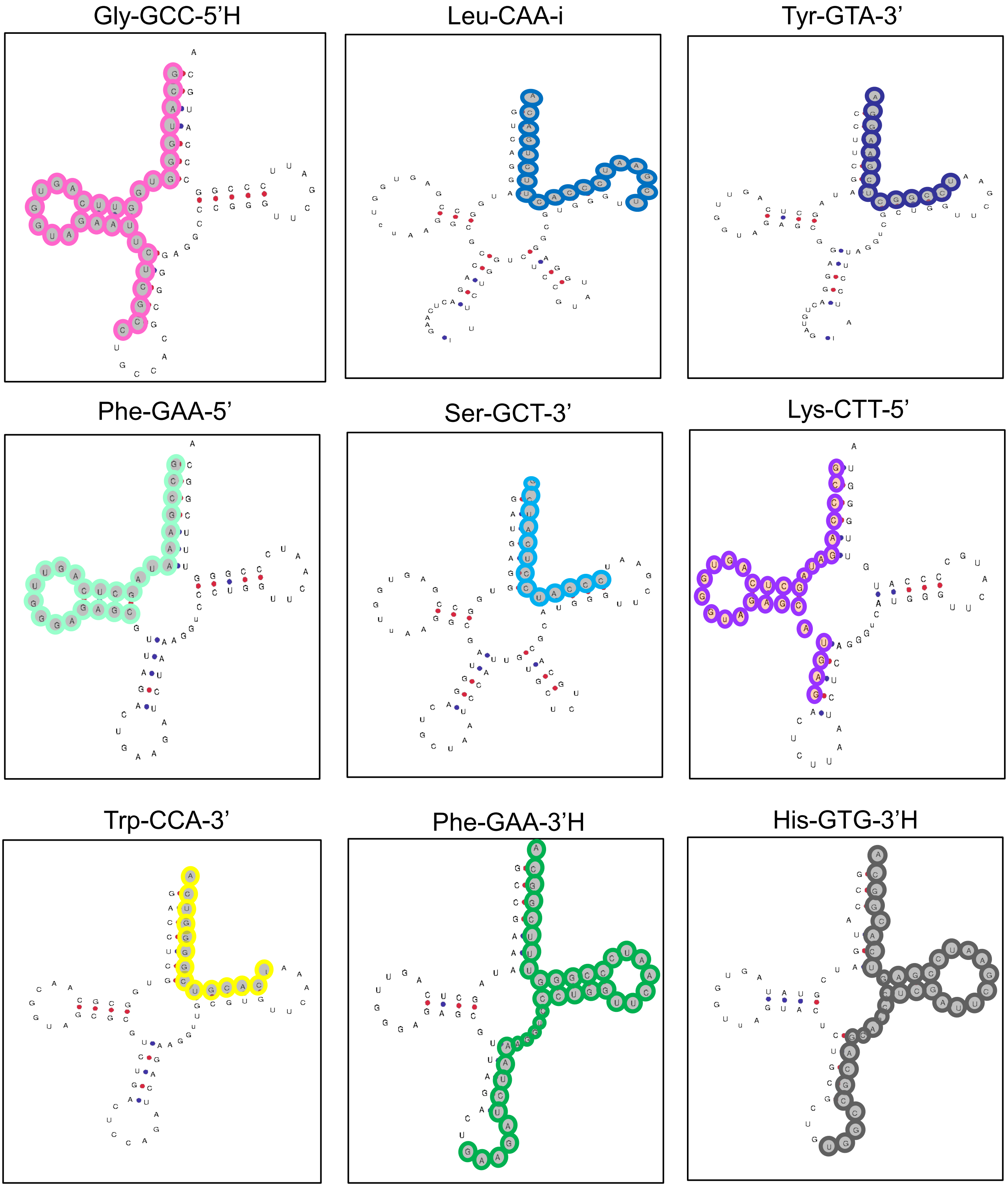

ESM Figure 2B: Selected genomic tRNA-derived fragments displaying changes in NOD islets during insulitis. The selected tRFs are aligned to the secondary structure of the tRNAs of origin. The indicated tRFs represent the sequences amplified by qPCR in Fig. 1C.

Adapted from: http://gtrnadb.ucsc.edu/genomes/eukaryota/Mmusc10/Mmusc10-displayed-gene-list.html

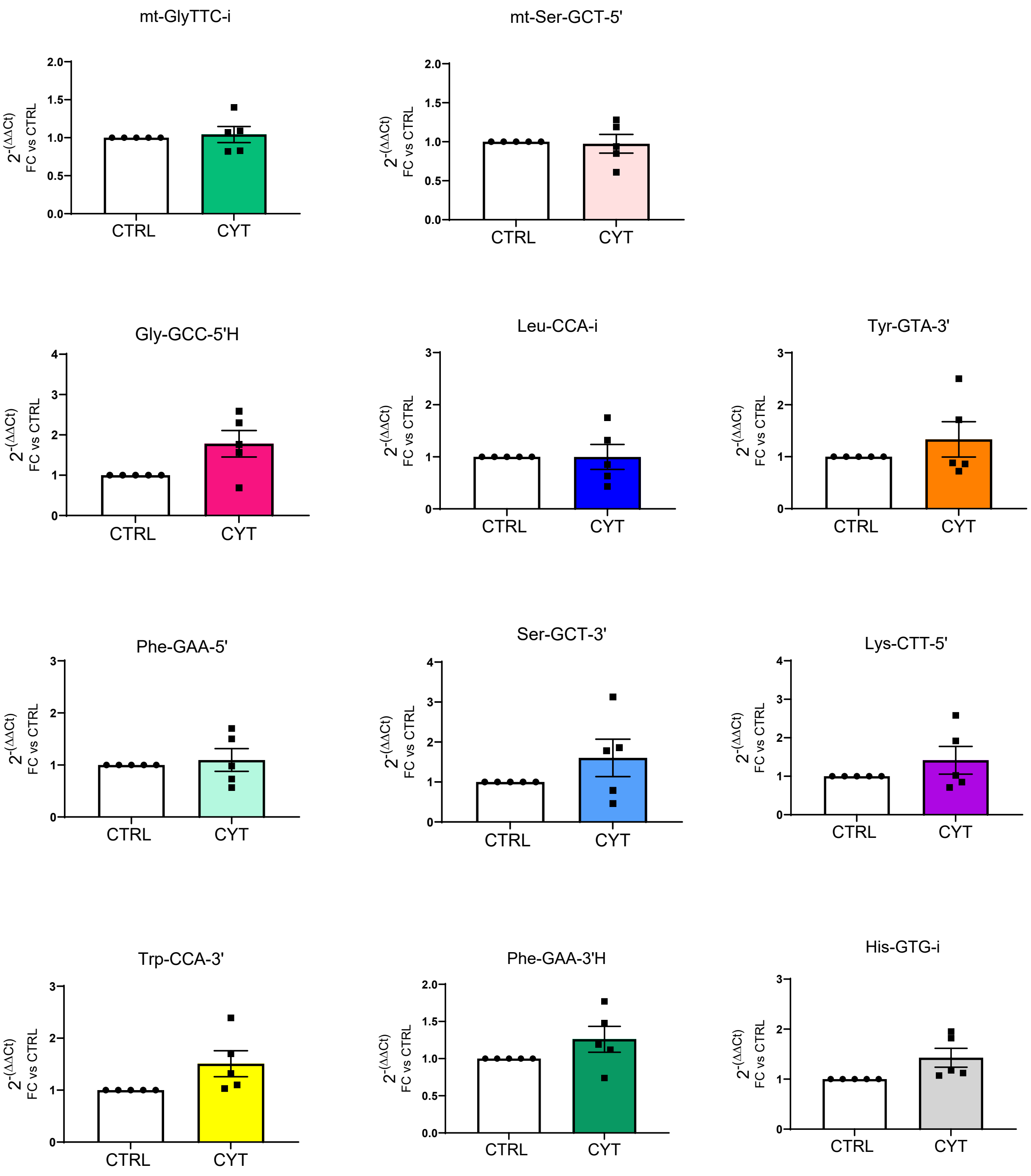

ESM Figure 3: Effect of proinflammatory on human tRFs. Human islet cells were incubated in the absence (Ctrl) or in the presence of a mix of cytokines (IL-1β, IFN-γ and TNF-α) for 24h. The level of the indicated tRFs determined by RT-qPCR. The data are shown as fold changes vs CTRL (n = 5). The level of human mt-Met-CAT was below detection limit and is not presented.

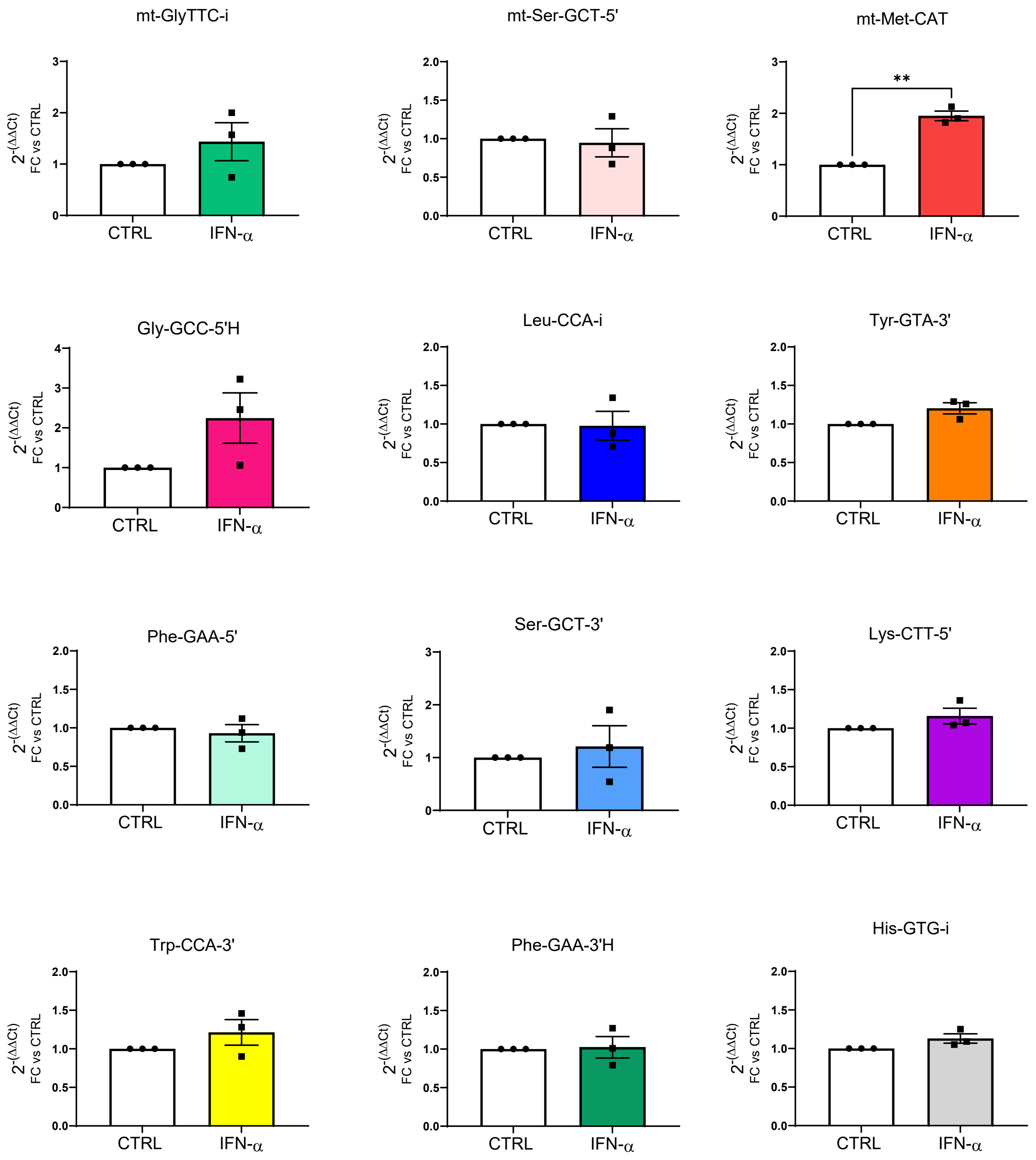

ESM Figure 4: Effect of IFNα on the level of the indicated tRFs. Mouse pancreatic islets were incubated for 24h in the presence or the absence of IFNα . The level of the indicated tRFs was assessed by RT-qPCR. The data are shown as fold changes vs control (n = 3). **$p < 0.01$, Ratio Paired t-test.

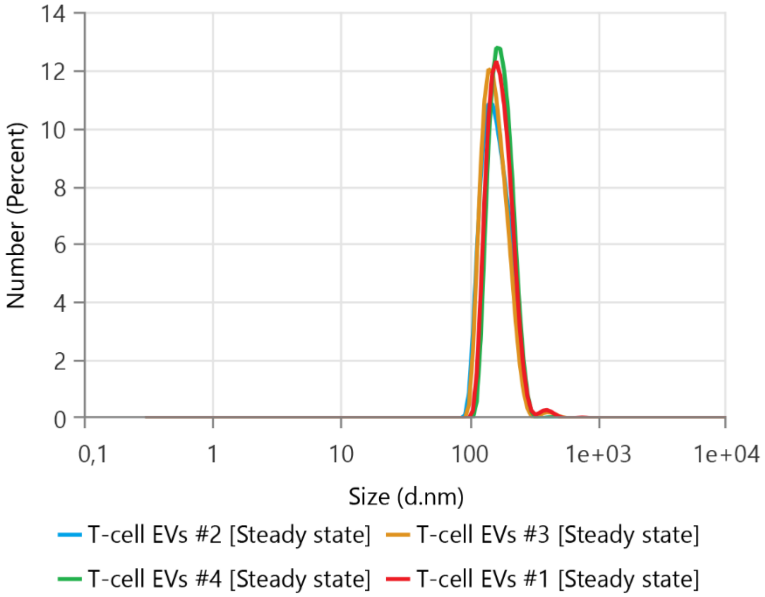

NOD T-cell EVs analyzed on Zetasizer
Summary of the size (main peak)

	Mean (nm)	Min (nm)	Max (nm)
EVs #1	162	99	325
EVs #2	151	93	305
EVs #3	141	86	303
EVs #4	165	106	326

ESM Figure 5: Characterization of the extracellular vesicles of CD4$^+$ T cells of NOD mice. Extracellular vesicles were isolated from the culture media of CD4+ T cells from NOD mice by sequential centrifugations as described in the methods. Vesicle size of four different preparations was assessed by Zetasizer.

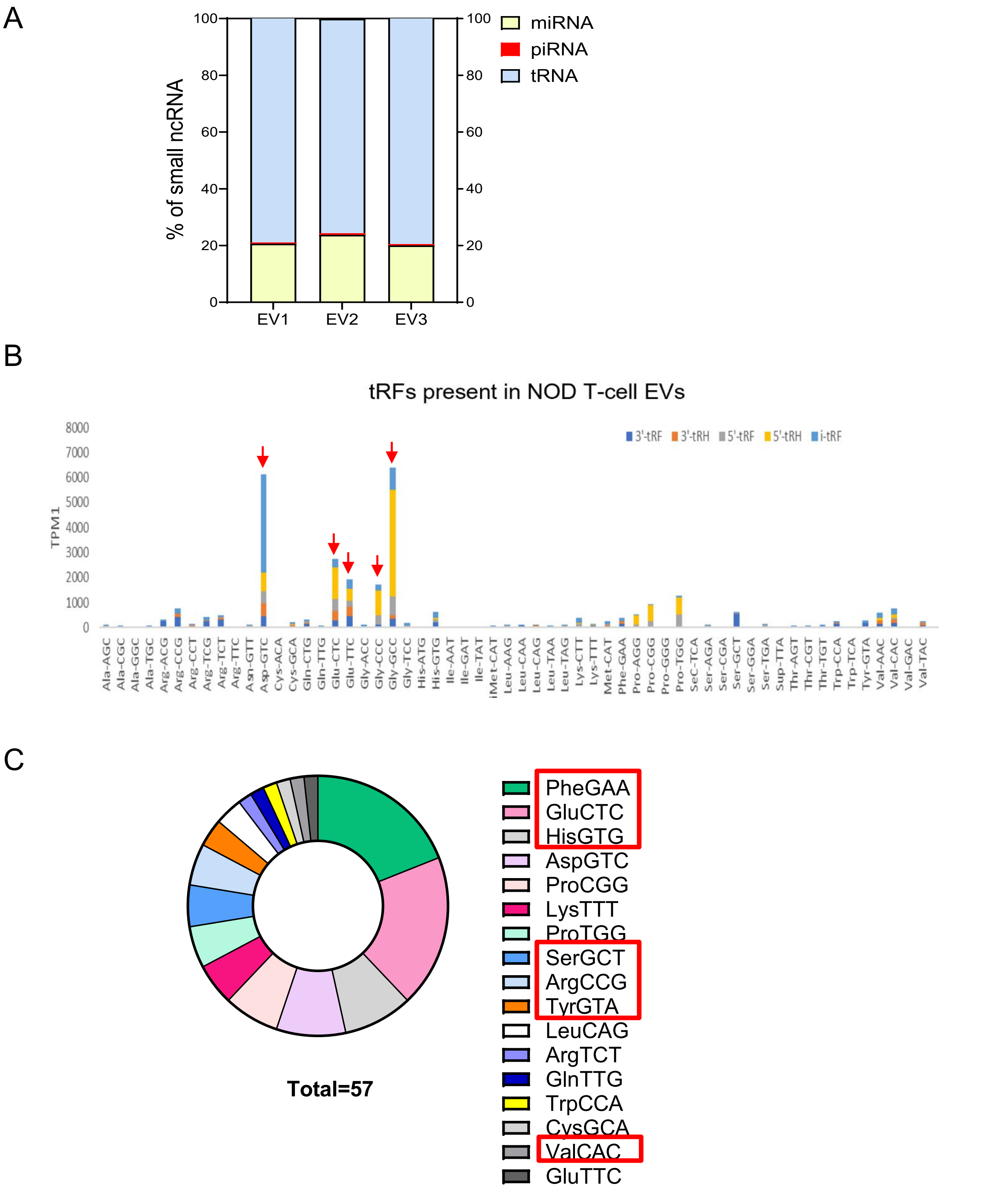

ESM Figure 6. T-cell EVs contain tRFs that are transferred to islet cells, modifying their tRFs pool. (A) Stacked bar graph representing the small RNA profile of EVs produced by activated CD4^{+}/CD25^{-} T-cells. Three individual experiments are represented. **(B)** Stacked plot describing the accumulation for five categories of tRFs (3'-tRF, 3'-tRH, 5'-tRF, 5'-tRH and i-tRF) originating from the same isodecoder tRNA. The average of TPM1 value and SD of the 3 exosomal samples were used. Red arrows indicate the five most abundant isodecoders. **(C)** Donut graph grouping the up-regulated tRFs in EV-treated mouse islet cells based on the isodecoder tRNA of origin. tRFs up-regulated in the islets of 8 weeks old NOD mouse are highlighted by red squares.

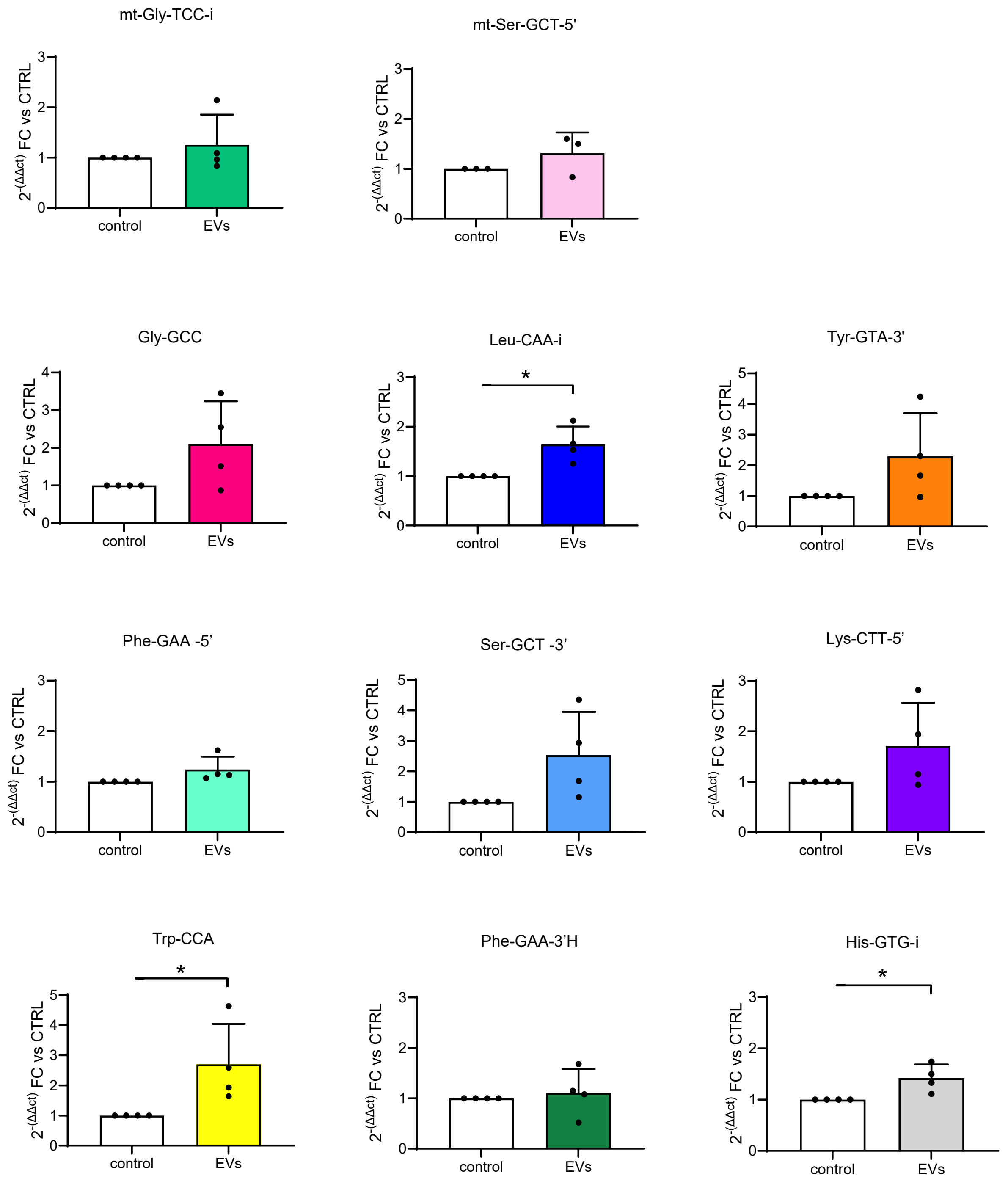

ESM Figure 7: EV-mediated transfer of tRFs from human CD4^{+} T cells to human islet cells. Human islet cells were incubated for 72h in the absence (control) or in the presence of EVs released from human CD4^{+} T lymphocytes. The samples previously used to assess the transfer of microRNAs (Guay et al. Cell metabolism 29, 348-361.e6, 2019) were analyzed to verify the transfer of tRFs. The level of the indicated tRFs was determined by RT-qPCR. The data are shown as fold changes vs control (n = 3-4). $*p < 0.05$, Ratio Paired t-test. The level of human mt-Met-CAT was below detection limit and is not presented.

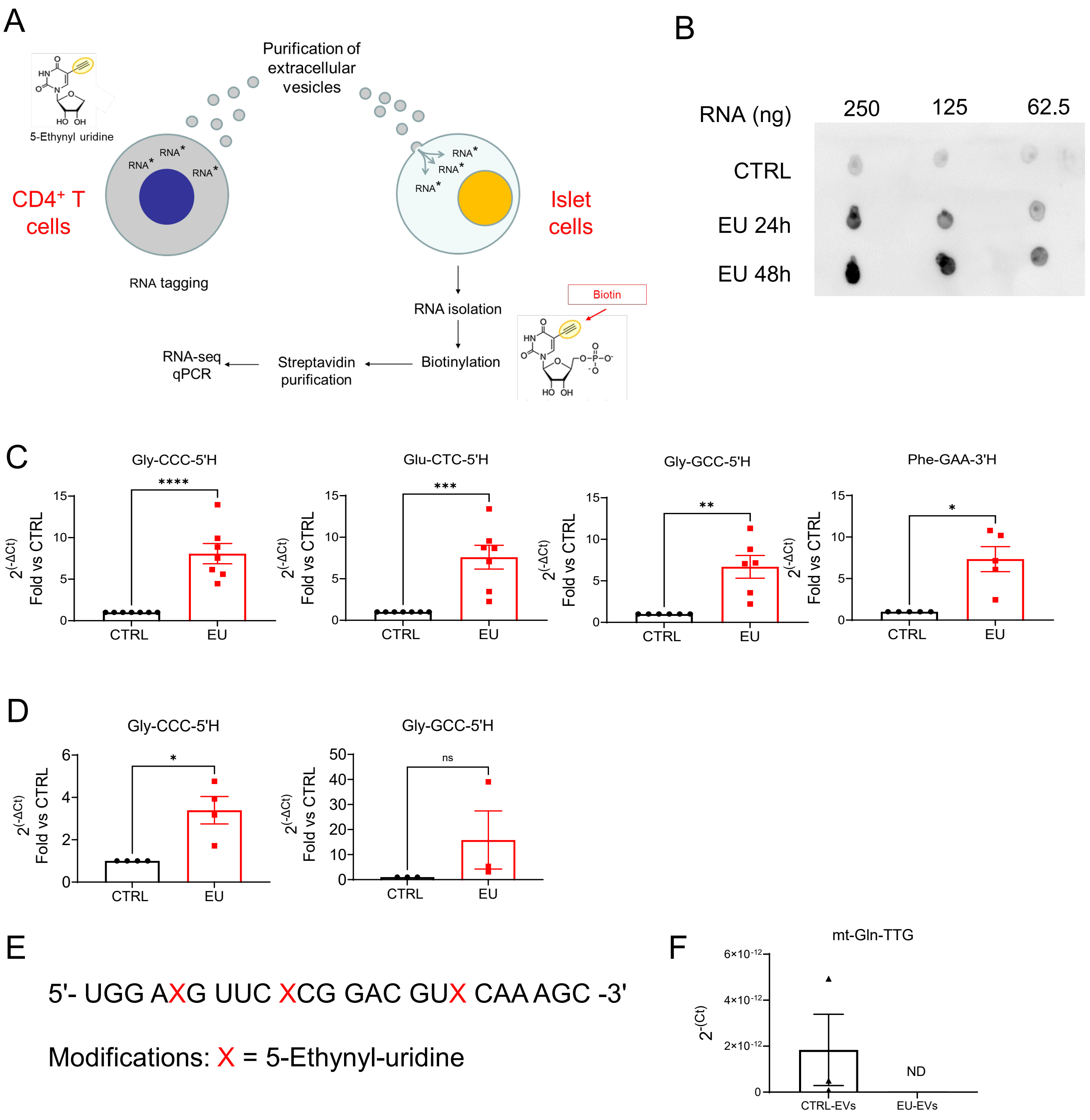

ESM Figure 8. T-cell RNA tagging with Ethynyl Uridine. **(A)** Schematic representation of the RNA-tagging approach: 5-Ethynyl Uridine (EU) is added to the culture medium at the concentration of 200 µM. Inside the cells, EU is converted in 5-Ethynyluracile mono phosphate and incorporated into nascent RNA. EU-tagged small non-coding RNAs (RNA*) are packaged into vesicles that are released in the extracellular space and captured by recipient cells (β-cells). EU-tagged RNA transferred to β-cells can be biotinylated, pulled-down using streptavidin beads and detected by qPCR or RNA-sequencing. **(B)** Dot-blot membrane loaded with biotinylated RNA from T-cells incubated with or without (CTRL) 200 µM EU for 24h (EU 24h), 48h (EU 48h). Different amounts of RNA were loaded, as indicated. **(C)** qPCR of the RNA recovered on streptavidin beads from EU-treated (EU) and untreated (CTRL) T-cells. **(D)**. EVs were isolated from EU-treated (EU) and untreated (CTRL) T-cells. The tagged RNA present in the EVs was purified on streptavidin beads and analyzed by Real-time PCR. Values are expressed in FC vs CTRL. (n =3-7). *$p < 0.05$, **$p < 0.01$, ***$p < 0.001$, and ****$p < 0.0001$ Ratio Paired T-test. **(E)** Sequence of the EU-containing spike-in oligonucleotide derived from C. elegans miR-238. **(F)** Mouse islet cells were incubated with T-cell EVs from untreated (CTRL) and EU-treated (EU) T-cells. RNA was extracted after 24h, biotinylated, purified on streptavidin beads and the level of mt-Gln-TTG analyzed by qPCR.

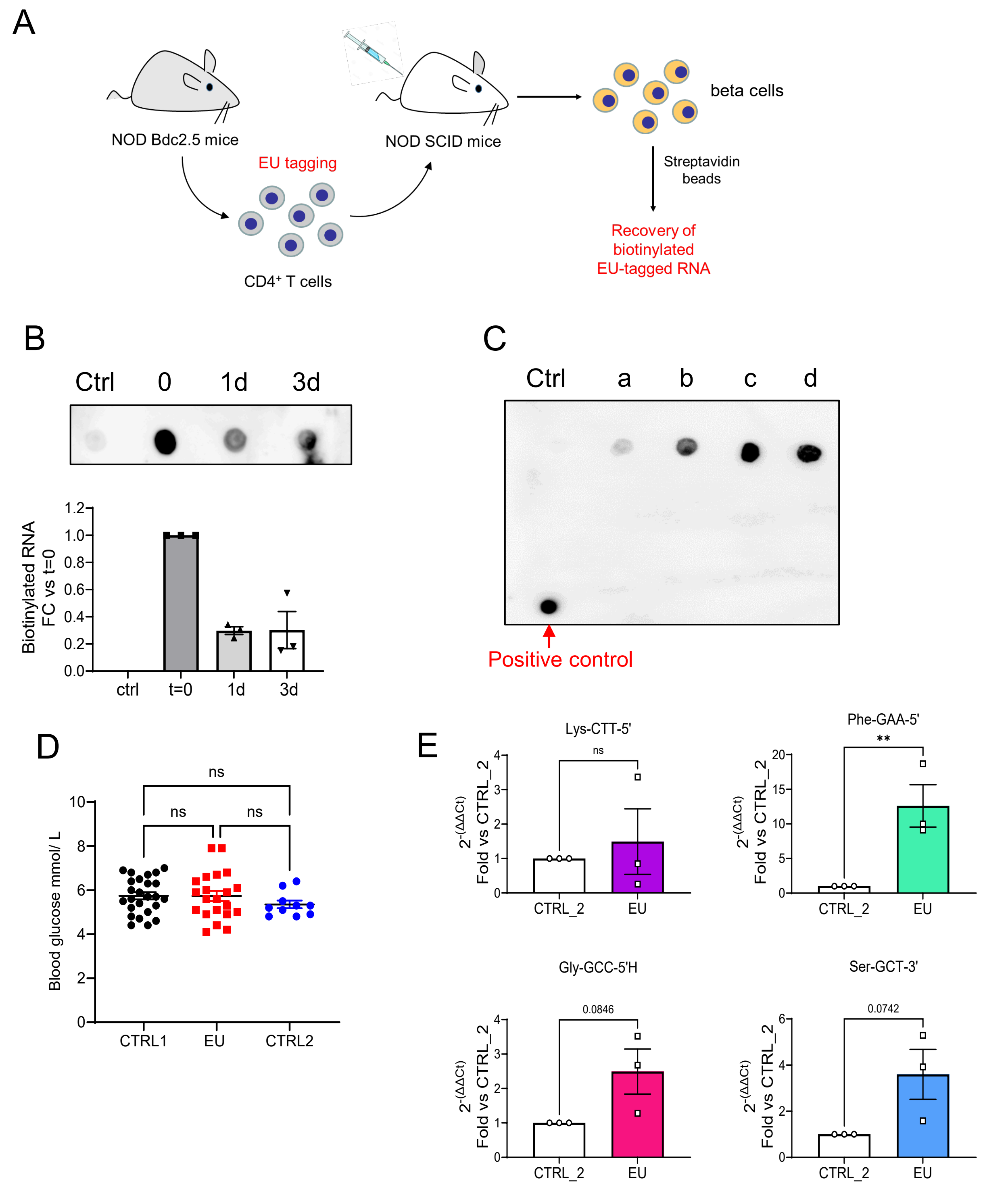

ESM Figure 9. tRFs directly transferred from CD4$^+$/CD25$^-$ T-cells during autoimmune diabetes. (A) Schematic representation of the in vivo tagging approach. **(B)** Dot-blot analysis and quantification of total EU-RNA present in CD4$^+$/CD25$^-$ T-cells after a 48h-pulse with 200 µM EU and in control conditions W/O EU (Ctrl). After EU incubation (t=0), the cells were washed, plated in fresh medium without EU, and RNA was collected at several time points as indicated (n=3). (**C**) Dot blot of EU-tagged RNA in NOD.BDC2.5 T-cells before injection in NOD.SCID mice (n=4). Ctrl= NOD.BDC2.5 T-cells not treated with EU. In lane «a» the amount loaded was ¼ compared to the other replicates (Ctrl, b, c, and d). A biotinylated RNA was spotted as a positive control (red arrow). (**D**) Glycemia measured on recipient NOD.SCID mice 48h after the injection of NOD.BDC2.5 T-cells. Mice were injected with saline solution (CTRL_1), with untreated T-cells (CTRL_2) and with EU-treated T-cells (EU). (**E**) Real-time PCR on the pull-down from FACS-sorted β-cells from NOD.SCID injected with EU-tagged T-cells (EU), compared to β-cells from NOD.SCID mice injected with not-treated T-cells (CTRL_2). EU-containing spiked-in C. elegans miR-238 was used to normalize the data (n =4). $^{**}p < 0.01$ by two-tailed unpaired t test.

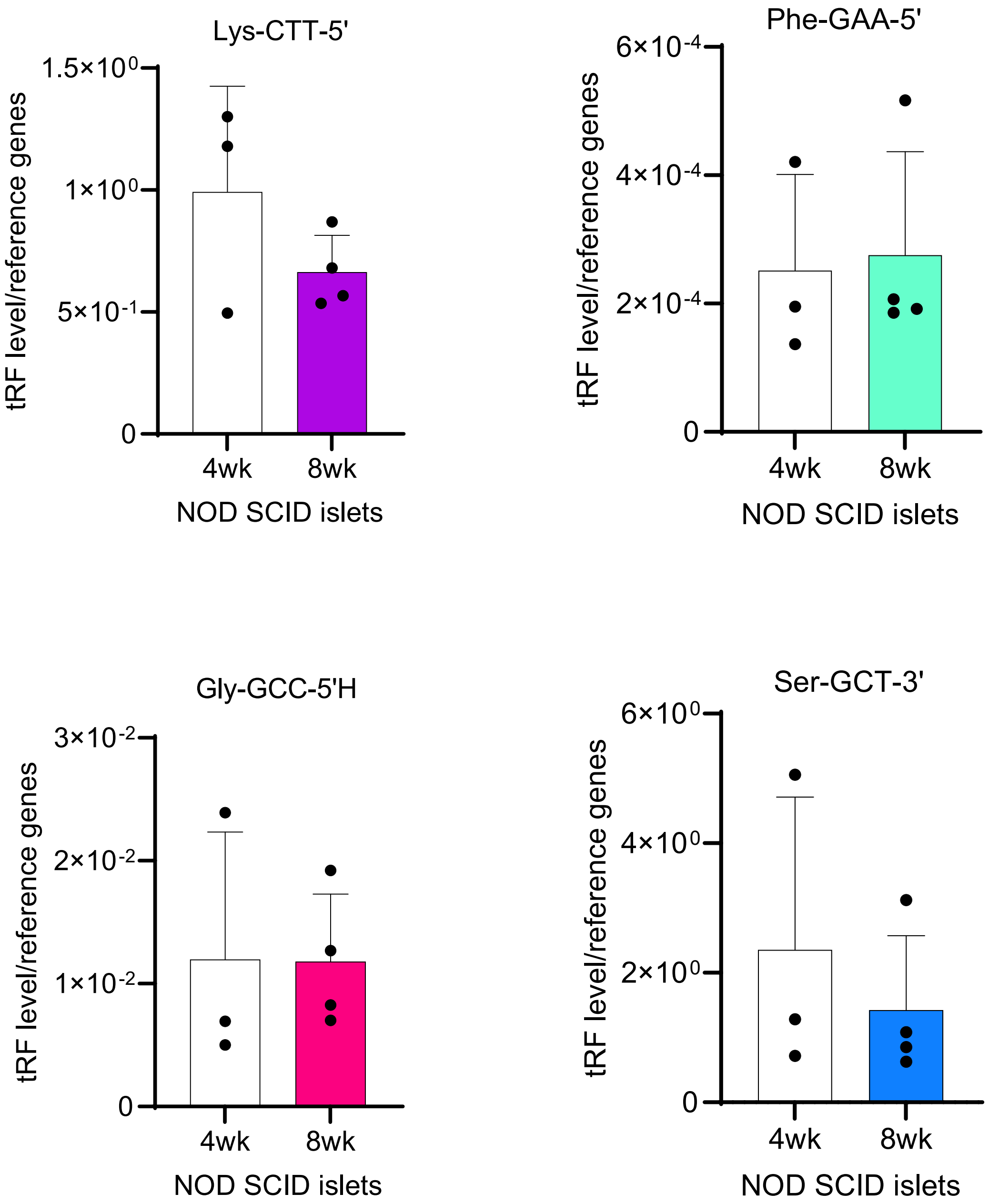

ESM Figure 10. Level of selected tRFs in the islets of NOD-SCID mice. The level of the indicated tRFs in the pancreatic islets of 4 and 8 weeks-old NOD SCID mice was measured by RT-PCR and was normalized to two reference small RNAs, Let7a and Let7f. Each point corresponds to a pool of the islets of 6 mice for 4 weeks-old animals and a pool of the islets of 3 mice for 8 weeks-old animals.

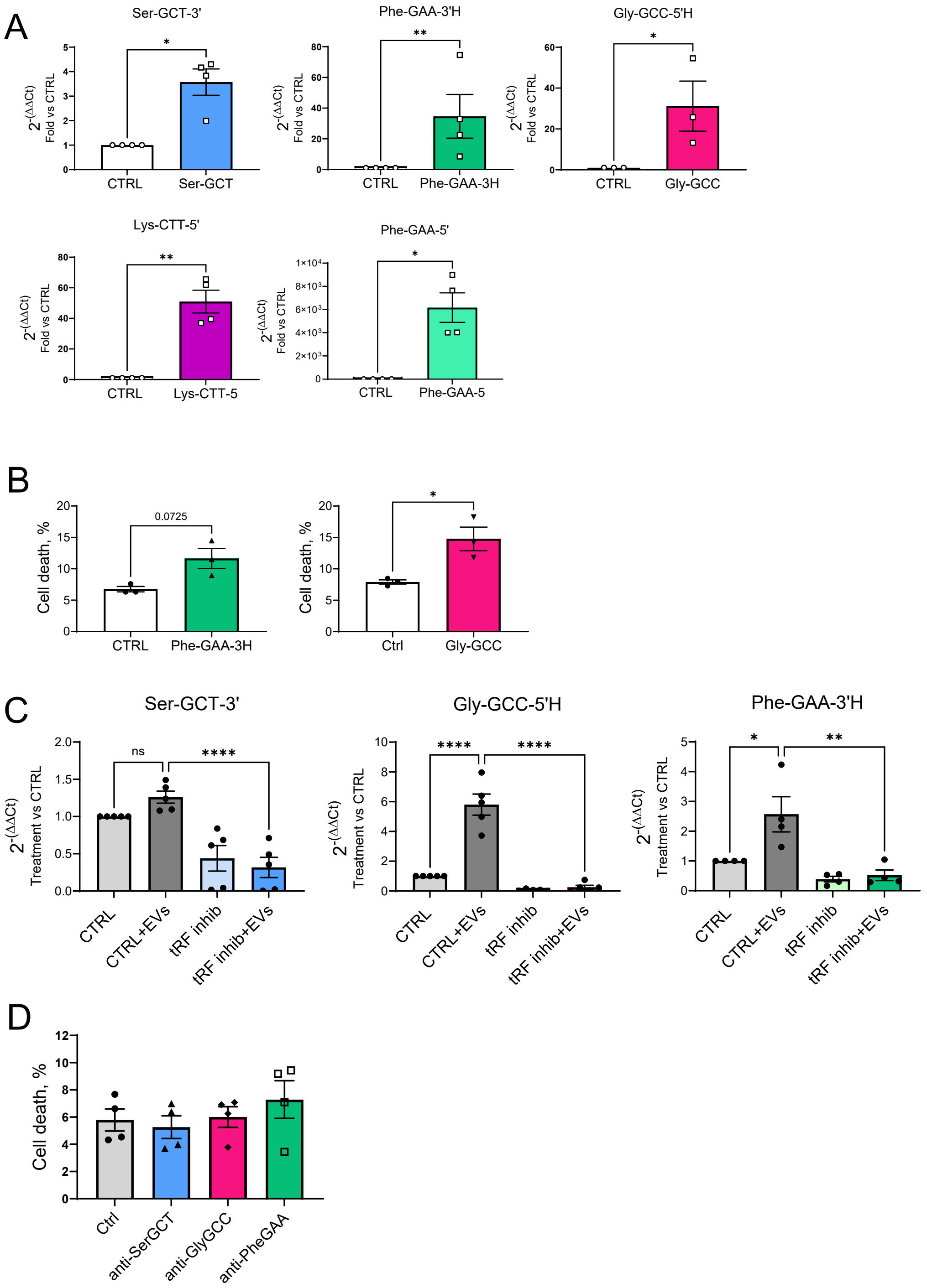

ESM Figure 11. Overexpression and inhibition of selected tRFs affects β-cell apoptosis. (**A** and **B**) Mouse islet β-cells were transfected with the indicated tRF mimics or a scramble sequence (CTRL). After 48h RNA was collected and tRF expression measured by qPCR (**A**). Islet cell death was assessed by scoring the cells displaying pycnotic nuclei upon Hoechst/ Propidium Iodate staining (**B**). (A and B) * $p < 0.05$, ** $p < 0.01$, Ratio paired T-test.

(**C**) Mouse islet cells were transfected with the indicated tRF inhibitors or a negative control (CTRL). After 24h, the cells were incubated with (EVs) or without (CTRL) T-cell EVs for 48h. RNA was collected and tRF expression measured by qPCR.

(D) Mouse islet cells were transfected with a negative control inhibitor (Ctrl) or with the indicated tRF inhibitors. Islet cell death was assessed by scoring the cells displaying pycnotic nuclei upon (H/PI) staining (around 5000 cells were counted per condition). (C and D) * $p < 0.05$, ** $p < 0.01$, **** $p < 0.0001$, One-way Anova, n=4-5.
